# Species distribution modelling of Bryde’s whales, humpback whales, southern right whales, and sperm whales in the southern African region to inform their conservation in expanding economies

**DOI:** 10.7717/peerj.9997

**Published:** 2020-09-22

**Authors:** Jean Purdon, Fannie W. Shabangu, Dawit Yemane, Marc Pienaar, Michael J. Somers, Ken Findlay

**Affiliations:** 1 Whale Unit, Mammal Research Institute, Department of Zoology and Entomology, University of Pretoria, Pretoria, South Africa; 2 Fisheries Management Branch, Department of Environment, Forestry and Fisheries, Cape Town, South Africa; 3 University of Cape Town, Marine Research Institute, Cape Town, Western Cape, South Africa; 4 uLwazi Node, South African Environmental Observation Network, Pretoria, South Africa; 5 Mammal Research Institute, Centre for Invasion Biology, Department of Zoology and Entomology, University of Pretoria, Pretoria, South Africa; 6 Centre for Sustainable Oceans, Cape Peninsula University of Technology, Cape Town, South Africa

**Keywords:** Cetaceans, Ensemble models, Important marine mammal areas, South Africa, Species distribution models

## Abstract

In southern African waters, information about species distribution and habitat preferences of many cetacean species is limited, despite the recent economic growth that may affect them. We determined the relative importance of eight environmental variables (bathymetry, distance to shore, slope, chlorophyll-a, salinity, eastwards sea water velocity, northwards sea water velocity and sea surface temperature) as drivers of seasonal habitat preferences of Bryde’s whales (*Balaenoptera brydei*), humpback whales (*Megaptera novaeangliae*), southern right whales (*Eubalaena australis*) and sperm whales (*Physeter macrocephalus*). Using presence only data from multiple sources, we constructed predictive species distribution models (SDMs) consisting of ensembles of seven algorithms for these species during both summer and winter. Predicted distribution for all cetaceans was high in southern Africa and, in particular, within the South African Exclusive Economic Zone (EEZ). Predictive models indicated a more pronounced seasonal variation for humpback, sperm and southern right whales than for Bryde’s whales. Southern right whales occurred closer to shore during winter, humpback whales were more likely to occur along the east coast in winter and the west coast in summer, and sperm whales were more concentrated off the shelf in winter. Our study shows that ensemble models using historical, incidental and scientific data, in conjunction with modern environmental variables, can provide baseline knowledge on important environmental drivers of cetacean distribution for conservation purposes. Results of this study can further be used to help develop marine spatial plans and identify important marine mammal areas.

## Introduction

Worldwide, cetaceans are increasingly threatened by anthropogenic activities (Williams et al., 2014; Braulik et al., 2017). These activities are likely to increase in South Africa with the initiation of Operation Phakisa (Harris et al., 2018; Purdon et al., 2020b). Operation Phakisa is a South African government initiative created to unlock the economic potential of the country’s oceans through increasing industry in five focal areas: marine transport and manufacturing; offshore oil and gas exploration; aquaculture; tourism and small harbour development and infrastructure (Van Wyk, 2015; Findlay, 2018). Understanding the potential impacts of these developing industries on cetaceans in South Africa requires a detailed understanding of cetacean distribution (Waggitt et al., 2020; Purdon et al., 2020b).

In order to protect cetaceans, information about their distribution is essential for their protection (Hoyt, 2012; Pennino et al., 2017). One way to get this information is to use species distribution models (SDMs), which are fitted using environmental biotic and abiotic factors to define distribution patterns (Bucklin et al., 2015; Breen et al., 2016). SDMs have been used extensively in the terrestrial environment and are slowly gaining recognition in the marine research environment (Redfern et al., 2006; Gregr et al., 2014; Reisinger et al., 2018; Waggitt et al., 2020). Modelling cetacean distribution is challenging because of the dynamic nature of ocean environments, the often highly mobile nature of cetacean species, and the difficulty and expense of obtaining adequate species distribution information (Gregr & Trites, 2001; Redfern et al., 2006). This has led to the development of SDMs based on data from several sources (Torres et al., 2013; Waggitt et al., 2020). The use of such datasets has two important challenges, the first being that the data are often spatially biased (Aiello-Lammens et al., 2015) and the second being that absence data are seldom available (Hirzel et al., 2006; Purdon et al., 2020a).

For SDMs to accurately predict species distribution, they require unbiased presence and absence data (Redfern et al., 2006) but such data are not always available. Several papers describe the most suitable methods to account for spatially biased data (Barbet-Massin et al., 2012; Aiello-Lammens et al., 2015; Stolar & Nielsen, 2015; Merow et al., 2016) and how to select pseudo-absences where real absences are unavailable (e.g., Hirzel et al., 2006; Phillips et al., 2009; Wisz & Guisan, 2009; Barbet-Massin et al., 2012). Spatially thinning data is one such method used to account for spatial biases in data; it involves the removal of as few presence points as possible without altering the quality of the data (Aiello-Lammens et al., 2015; Schmitt et al., 2017). To account for the lack of absence data, Barbet-Massin et al. (2012) suggest that the structure of presence data and the type of models used to create the SDMs will play a role in the pseudo-absence selection process.

There are varying advantages and disadvantages in individual SDMs, with documented discrepancies obtained in their performance (e.g., Phillips et al., 2009; Thuiller et al., 2009; Bucklin et al., 2015). To address some of these uncertainties, ensemble modelling (which combines multiple SDMs) is proving to be a robust method (e.g., Coetzee et al., 2009; Oppel et al., 2012; Zanardo et al., 2017; Pereira et al., 2018). The scope of ensemble modelling is relatively new in the marine environment with only a few studies being carried out on cetaceans (Zanardo et al., 2017; Purdon et al., 2020a).

There have been limited studies on the distribution of cetaceans in South Africa (e.g., Findlay, 1989; Findlay et al., 1992; Best, 2007). Given the sparsity of data related to cetacean sightings, the existing data need to be supplemented and updated so as to determine areas that could protect cetaceans from the potential threats of ocean economy development. Operation Phakisa aims to balance economic growth and environmental integrity (Sink, 2016) in the oceans, through a dedicated Marine Protection Services and Governance initiative, that includes the recently (23 May 2019) approved marine protected areas (MPAs) network (RSA, 2019) and the promulgation of a Marine Spatial Planning Act. Within the MPAs, only one, the seasonal Walker Bay whale sanctuary, was established for cetacean conservation. It is aimed at protecting southern right whales during their breeding season, despite this not being the primary calving ground of the species. Considering South Africa’s high cetacean diversity (Findlay et al., 1992; Best, 2007), the low number of MPAs directly protecting them is a concern (Purdon et al., 2020a).

We selected four large commonly encountered whale species in South African waters: Bryde’s whales (*Balaenoptera brydei,* where two forms are found), humpback whales (*Megaptera novaeangliae*), southern right whales (*Eubalaena australis*) and sperm whales (*Physeter macrocephalus*) for this study in accordance with the following rationale. (1) Their presence data are sufficient to construct SDMs. (2) They were harvested almost to extinction during the whaling era (Hoyt, 2012) (Bryde’s whale to a lesser extent), and today, despite the international moratorium on commercial whaling initiated in 1985/1986, they face new anthropogenic threats that could affect their recovery (Purdon et al., 2020b). Humpback whales and southern right whales are recovering at about 10% and 7% a year, respectively. Bryde’s whales and sperm whales are data deficient in this regard (Best, 2007). (3) These cetacean species are an important source of income for the tourism industry, with the South African whale watching industry bringing in 105 million Rand in 2014 (Duncan et al., 2016). (4) South Africa offers the required habitats for important life stages for each of these four cetaceans (Best, 2007).

In this study, we model the seasonal distribution of four large commonly-encountered whale species in southern African waters using an ensemble of SDMs with presence only data. The results of this study will help to identify potential habitats of these four species. It will enable policy makers, conservationists and managers to better conserve and protect these cetaceans from anthropogenic activities associated with the expansion of the South African ocean economy proposed under Operation Phakisa.

## Methods

### Study area

**Figure 1:**
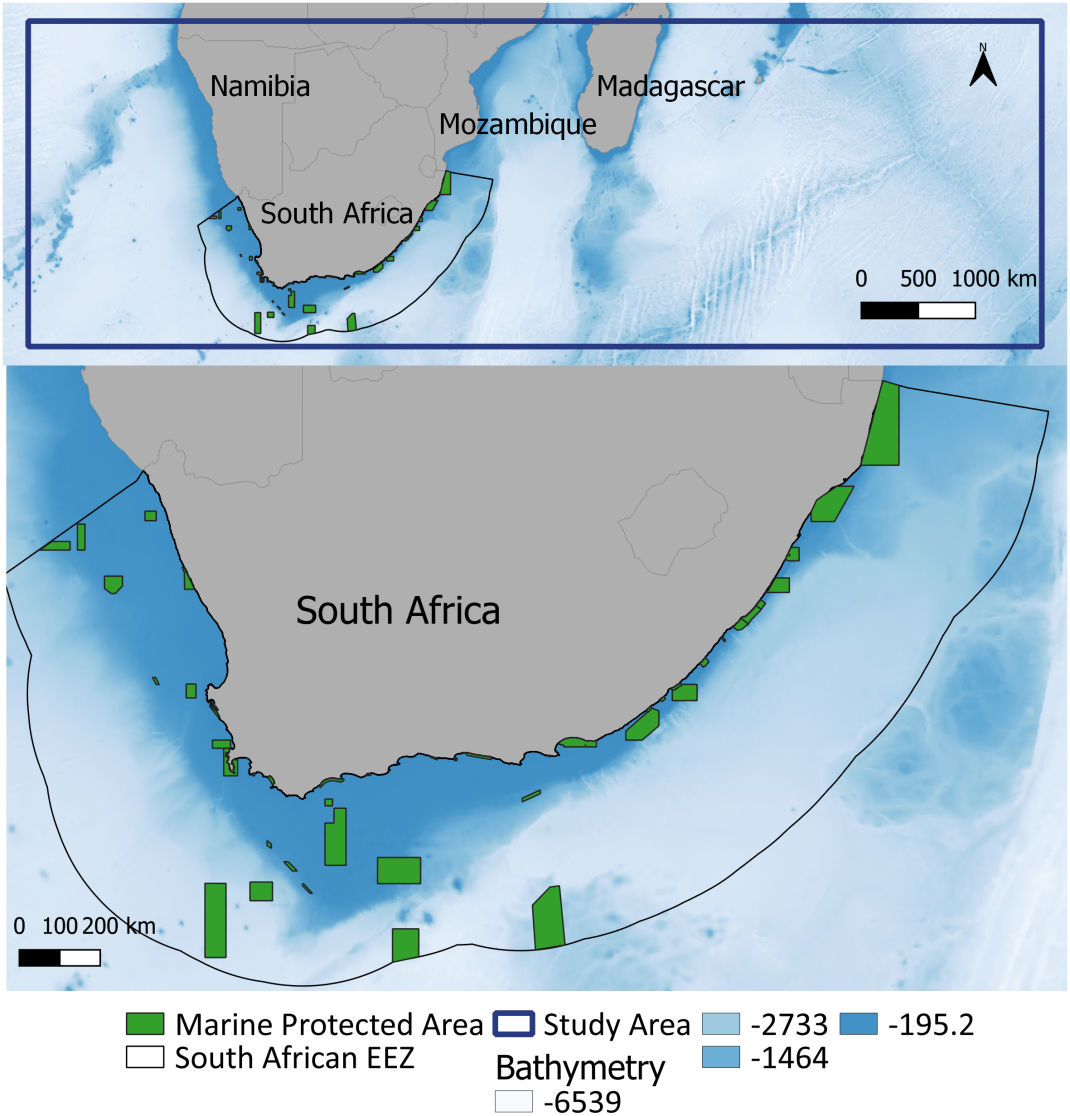
Study area within the southern African region (top: delineated by the blue line) and the South African EEZ and associated MPAs magnified. Maps plotted using QGIS (https://www.qgis.org/en/site/). Bathymetry is from GEBCO (GEBCO Compilation Group, 2019).

The study area (limited by the co-ordinates 16°S, 0°E to 38°S, 80°E) encompasses the western Indian Ocean and the southeast Atlantic Ocean (Fig. 1), covering both cold and warm water habitats of these cetaceans (Findlay et al., 1992; Best, 2007). We chose this area to include all the available information on species sightings and to determine habitats suitable for whale distribution within the area.

### Presence data

**Figure 2:**
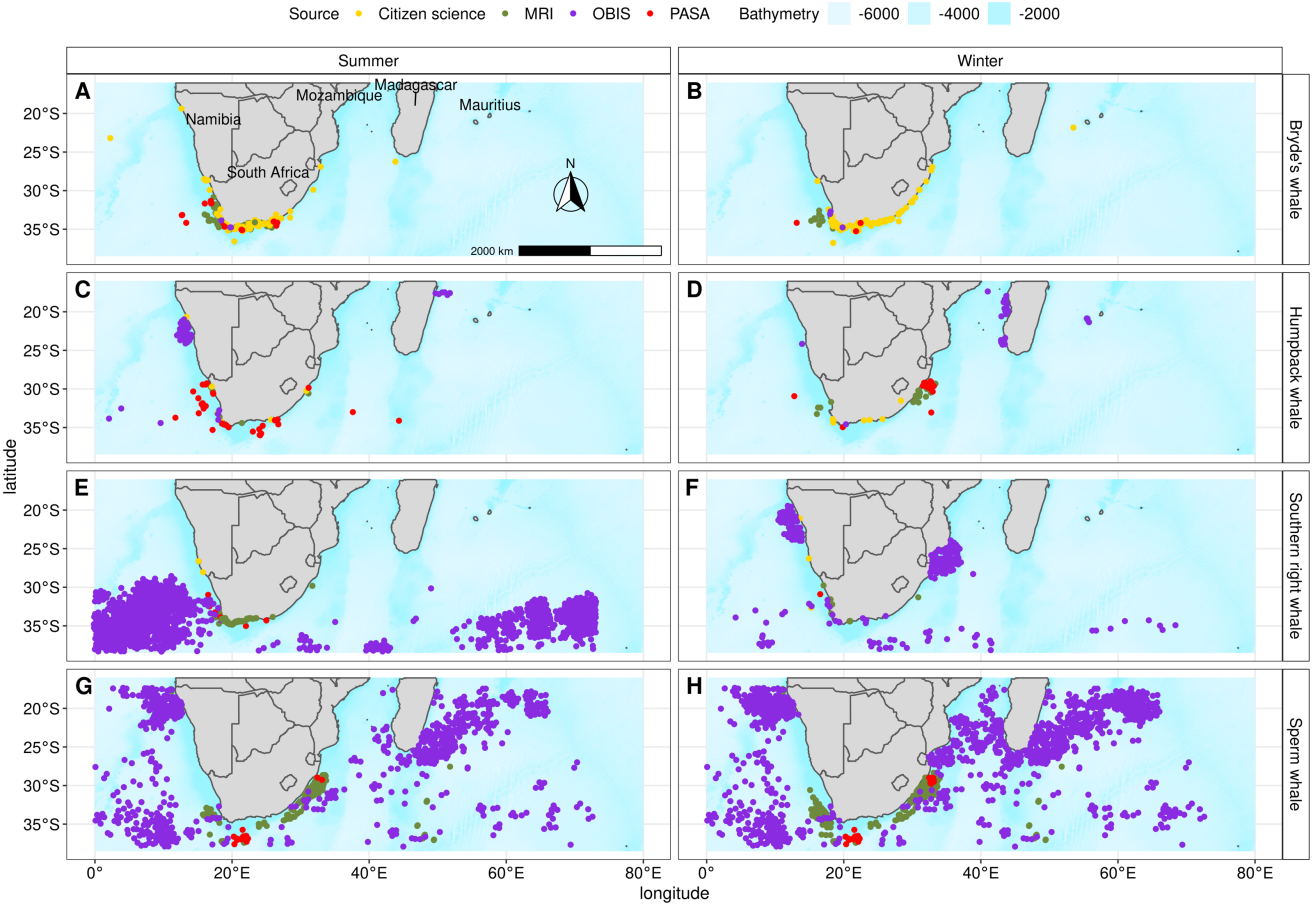
Presence points and their source for Bryde’s whale, humpback whale, southern right whale and sperm whale during summer and winter that were used in the individual algorithms and the ensemble model. Bathymetry is from GEBCO (GEBCO Compilation Group, 2019).

We collated data for all four species from opportunistic and dedicated scientific surveys covering the period from 1913 to 2016 (Table 1, Fig. 2, Fig. S1 and Table S1). This specific period was used as it included as many presence points as possible and because data falling outside these dates were not available. The majority of the data we used originated from the Whale Unit of the Mammal Research Institute at the University of Pretoria (44%) and the Ocean Biogeographic Information System Spatial Ecological Analysis of Megavertebrate Populations (OBIS-SEAMAP) (47%) (Halpin et al., 2009). Incidental sightings (7%) (Nan Rice, CEO: Dolphin Action and Protection Group, Fish Hoek) and seismic survey marine mammal observations in South Africa (2%) (Petroleum Agency of South Africa, PASA) made up the rest of the dataset.

**Table 1:** Numbers of spatially thinned presence points, original presence points, and pseudo- absences used in the species distribution modelling of the Brydes whale, humpback whale, southern right whale and sperm whale during both seasons. ANN is Artificial Neural Network (Venables & Ripley, 2002), CTA is Classification Tree Analysis (De’ath & Fabricius, 2000), GBM is Generalized Boosting Model (Elith, Leathwick & Hastie, 2008), GLM is Generalized Linear Model (Lee & Nelder, 2001), MARS is Multivariate Adaptive Regression Splines (Friedman, 1991), RF is Random forest (Cutler et al., 2007), SVM is Support Vector Machines (Schmitt et al., 2017).

Algorithm acronym (algorithm type)	Presence and pseudo-absence data	**Bryde’s whale**		**Humpback whale**		**Southern right whale**		**Sperm whale**	
		**Summer**	**Winter**	**Summer**	**Winter**	**Summer**	**Winter**	**Summer**	**Winter**
ANN (Machine learning)	Presence points	210	100	81	75	1,219	202	908	1,789
	Pseudo-absence	210	82	81	59	1,219	157	908	1419
CTA (Classification)	Presence points	210	100	81	75	1219	202	908	1,789
	Pseudo-absence	210	88	81	62	1,219	157	908	1,367
GBM (Regression)	Presence points	210	100	81	75	1219	202	908	1,789
	Pseudo-absence	210	74	81	61	1,219	162	908	1,390
GLM (Regression)	Presence points	210	100	81	75	1219	202	908	1,789
	Pseudo-absence	1,000	769	1,000	774	1,000	790	1,000	791
MARS (Regression)	Presence points	210	100	81	75	1219	202	908	1,789
	Pseudo-absence	1000	793	1,000	813	1,000	788	1,000	786
RF (Machine learning)	Presence points	210	100	81	75	1219	202	908	1,789
	Pseudo-absence	210	81	81	62	1219	152	908	1,398
SVM (Machine learning)	Presence points	210	100	81	75	1219	202	908	1,789
	Pseudo-absence	210	76	81	57	1219	168	908	1,414
**Original number of presence points**		**227**	**129**	**81**	**75**	**1,271**	**204**	**972**	**2,046**
Percentage contribution from each source	MRI	38	5	7	29	56	17	31	49
	OBIS	1	1	46	28	44	81	67	49
	Citizen Science	54	93	8	9	0	2	0	0
	PASA	7	1	39	34	0	0	2	2

### Environmental data

We selected environmental variables based on known ecological relationships between cetaceans and environmental variables (Table 2) (Best, 2007; Torres et al., 2013; Breen et al., 2016). Topographic variables included bathymetry (metres), distance to shore (kilometres) and slope (degrees). Oceanographic variables included sea surface temperature (SST in °C) chlorophyll-a concentration (chl-a in mg m^−3^), salinity (in psu), eastwards sea water velocity (U_o_ in m s^−1^) and westwards sea water velocity (V_o_ in m s^−1^) (Fig. S2). We used SST, salinity, U_o_ and V_o_ data derived from satellites. Wernand, Van de rWoerd & Gieskes (2013) have shown that there has been an increase of chl-a concentration in the Atlantic Ocean and a decrease in the Indian Ocean with no overall significant chl-a trends worldwide from 1989 to 2000. SST has risen between 1.2 °C and 1.6 °C in the high latitudes and between 0.4 °C and 1 °C in the tropics and sub-tropics over the last 100 years (Deser, Phillips & Alexander, 2010). Despite these changes, studies by Purdon et al. (2020a), Purdon et al. (2020b) and Torres, Read & Halpin (2016) used averages of oceanographic variables, suggesting that they provide sufficient information for modelling baseline cetacean distribution in conjunction with presence data that dates back to 1913. In this study we averaged chl-a seasonally over the period 2002 to 2016, and salinity, U_o,_ V_o_ and SST seasonally over the period 1991 to 2016 in their original projection (WGS 84 (EPSG 4326)) and at their origional spatial resolution (∼8 km) in ArcMap (Table 2, Esri, Redlands, CA, USA).

**Table 2:** The environmental variables used for species distribution models. MARSPEC = MARine SPatial ECology.

Environmental variable	Resolution	Source	Units	Reference
Bathymetry	30 arc-sec (∼1 km)	MARSPEC	Metres	http://www.marspec.org
Slope	30 arc-sec (∼1 km)	MARSPEC	Degrees	http://www.marspec.org
Distance to Shore	30 arc-sec (∼1 km)	MARSPEC	Kilometres	http://www.marspec.org
Chlorophyll-a (chl-a)	4 km	Copernicus - OCEANCOLOUR_GLO_CHL_L4_REP_OBSERVATIONS_009_082	mg m^−3^	(Volpe et al. 2019)
Sea surface temperature (SST)	0.083° (∼8 km)	Copernicus –GLOBAL_REANALYSIS_PHY_001_030	°C	(Fernandez & Lellouche 2018)
Salinity	0.083° (∼8 km)	Copernicus –GLOBAL_REANALYSIS_PHY_001_030	Psu	(Fernandez & Lellouche 2018)
Eastwards seawater velocity (V_o_)	0.083° (∼8 km)	Copernicus –GLOBAL_REANALYSIS_PHY_001_030	m s −1	(Fernandez & Lellouche 2018)
Northwards seawater velocity (U_o_)	0.083° (∼8 km)	Copernicus –GLOBAL_REANALYSIS_PHY_001_030	m s −1	(Fernandez & Lellouche 2018)

To ensure all variables maintained the same spatial resolution and projection, we resampled them to a 0.083° (∼8 km) grid using a nearest neighbour interpolation and re-projected them to a standard WGS84, EPSG 4326 geographic projection in ArcMap. They were then clipped to the study area extent using ArcMap. We evaluated multi-collinearity between environmental variables by testing the variable inflation factor (VIF) using the ‘car’ package (Fox & Weisberg, 2011) in R (R Core Team, 2020). We retained all variables as the VIF was less than three, indicating no multi-collinearity (O’Brien, 2007).

### Species distribution modelling

We used ensemble modelling implemented in the ‘SSDM’ package (Schmitt et al., 2017) within R to predict preferred species habitat using seven algorithms (Table 1). We used the algorithms as they consisted of different modelling approaches, including three regression methods, one classification method and three machine learning methods to build ensemble models for each species seasonally (Table 1). To determine seasonal variation, we followed Findlay (1989) in creating SDMs separately for summer (October to March) and winter (April to September) months.

The dataset contained both presence and absence data from scientific surveys, and marine mammal observers and passive acoustic monitors on oil and gas exploration surveys, and presence only records from opportunistic sightings. To compensate for the lack of effort data, we used pseudo-absences selected randomly in the study area (Fig. S1). The number of pseudo-absence points selected was based on the recommendations of Barbet-Massin et al. (2012) for the different types of algorithms used (Table 1). Barbet-Massin et al. (2012) suggest that, for classification and machine learning techniques, the same number of pseudo-absence points as presence points should be selected and averaged over several runs. For regression techniques, many pseudo-absence points (2% of the study area which is 25 218 188 km^2^ for our study) should be chosen, whereas for techniques such as multi adaptive regression splines and discriminant analysis, fewer pseudo-absence points should be chosen and both averaged over several runs. We selected the number of pseudo-absence points 10 times on a random basis using the ‘SSDM’ package default settings based on the recommendations of Barbet-Massin et al. (2012) (Table 1 and Fig. S1).

To account for spatially biased data, which are prone to spatial autocorrelation, we used spatial thinning within the ‘SSDM’ package (Schmitt et al., 2017). We evaluated the algorithms and ensemble model performance using the holdout method in the SSDM package, where the initial dataset was split into separate training (70% of the data) and evaluation (30% of the data) sets (Schmitt et al., 2017). This process was repeated 10 times. We used a standard evaluation metric, area under the receiver operating characteristic curve (AUC). Scores ranged between 0.5 and 1, with those close to 1 indicating that the model has excellent performance, whereas values around 0.5 denote models that are no better than random.

Variable importance was measured by determining how much the correlation changed between predicted values before and after permuting each variable.

\begin{eqnarray*}{I}_{v}=1-Cor({P}_{f},{P}_{v}) \end{eqnarray*}

where *I*_*v*_ is index of importance of a variable, *Cor* is correlation coefficient, *P*_*f*_ is prediction from the full model, *P*_*v*_ is prediction after permuting or reshuffling the variable *v*.

We computed the partial effects of each environmental variable by predicting the response for a specific variable while holding the other variables at their mean. For example, if bathymetry was the variable that best explained the cetacean species distribution, scores of the partial effects closer to one would indicate at what depths the cetaceans were likely to be found.

To create the final ensemble model, we calculated the average of all seven algorithms if their AUC scores were over 0.7. We projected the final ensemble models for each species and season (summer and winter) on to a predicted distribution map. Probability of occurrence ranged between zero and one, with one indicating the highest probability of species presence. We created uncertainty maps using the variance of predicted probability of occurrence among the algorithms, where areas of agreement are characterised by low variance amongst algorithms (Schmitt et al., 2017).

## Results

### Ensemble model and algorithm performance

**Figure 3:**
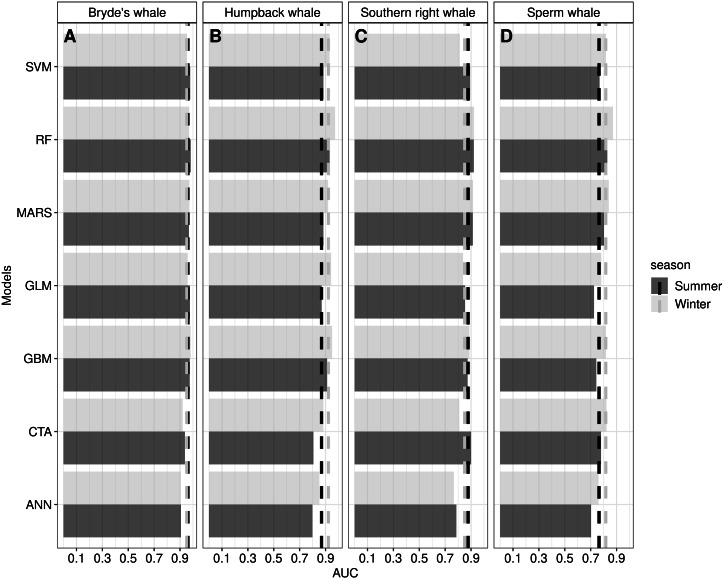
The Area Under the Curve (AUC) scores for the ensemble model (dashed lines) and seven individual algorithms for Bryde’s whale, humpback whale, southern right whale and sperm whale during the summer and winter season. AUC scores over 0.5 indicate that the algorithm performed better than random. ANN is Artificial Neural Network (Venables & Ripley, 2002), CTA is Classification Tree Analysis (De’ath & Fabricius, 2000), GBM is Generalized Boosting Model (Elith, Leathwick & Hastie, 2008), GLM is Generalized Linear Model (Lee & Nelder, 2001), MARS is Multivariate Adaptive Regression Splines (Friedman, 1991), RF is Random forest (Cutler et al., 2007), SVM is Support Vector Machines (Schmitt et al., 2017). The dashed line for Bryde’s whale ensemble model summer is overlaid by the dashed line for Bryde’s whale winter ensemble model.

Overall, we predicted that the ensemble models would produce higher AUC scores than the individual algorithms. Analysis of the results, however, indicates that, with the exception of artificial neural network, ANN, the performance of individual algorithms was equal to or better than the ensemble model (Fig. 3 and Table S2). The RF algorithm produced the highest AUC scores for all species in both seasons except for the Bryde’s whale in winter where the algorithm GBM produced the highest AUC score (Fig. 3 and Table S2).

### Species distribution modelling

**Figure 4:**
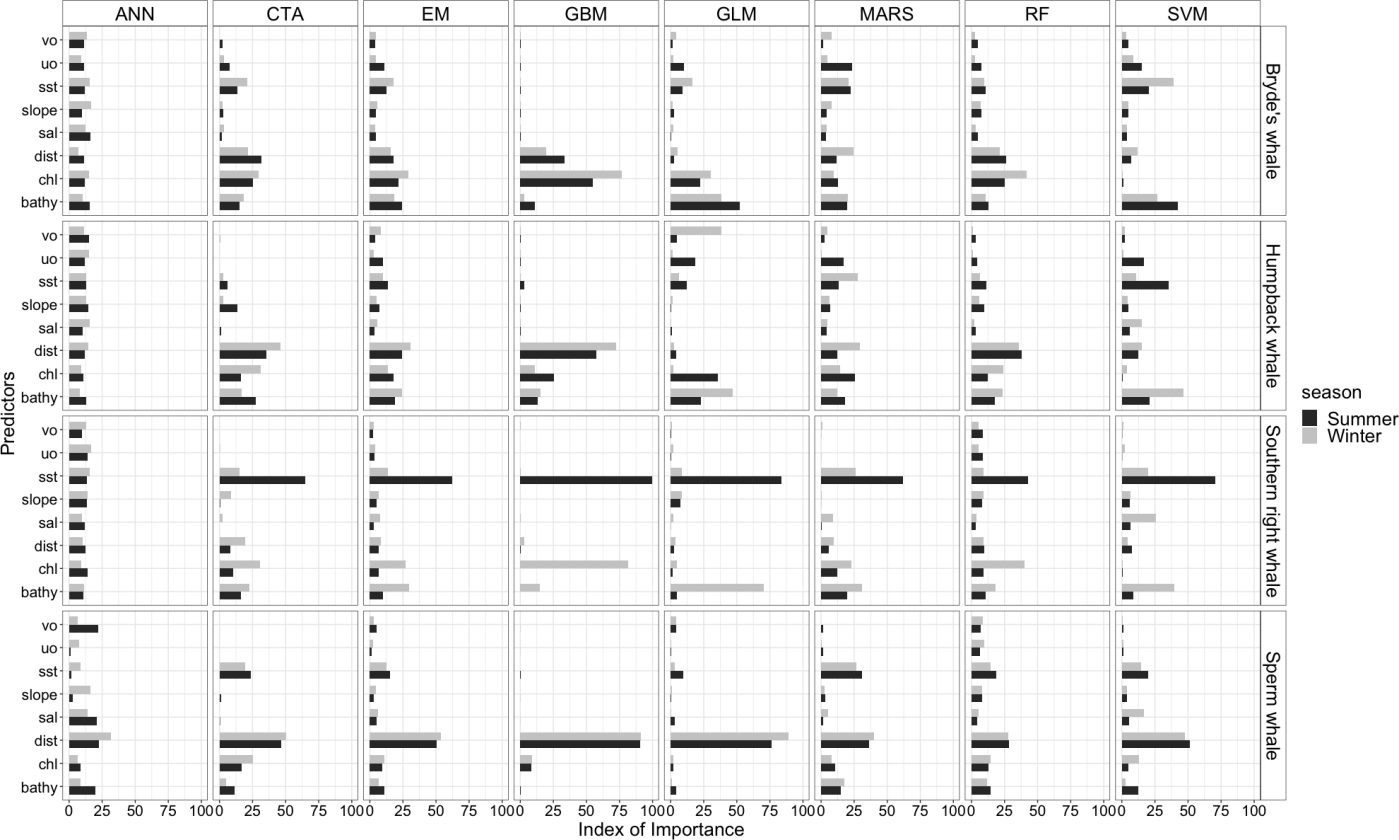
Index of relative importance of the eight predictors for each algorithm and the ensemble model (EM) for the Bryde’s whale, humpback whale, southern right whale and sperm whale during winter and summer. ANN is Artificial Neural Network (Venables & Ripley, 2002), CTA is Classification Tree Analysis (De’ath & Fabricius, 2000), GBM is Generalized Boosting Model (Elith, Leathwick & Hastie, 2008), GLM is Generalized Linear Model (Lee & Nelder, 2001), MARS is Multivariate Adaptive Regression Splines (Friedman, 1991), RF is Random forest (Cutler et al., 2007), SVM is Support Vector Machines (Schmitt et al., 2017). Bathy, bathymetry (m): Chl, chlorophyll-a concentration (mg m^−3^); Dist, distance to shore (km): Sal, salinity (psu); Slope, angle of slope (degrees); SST, sea surface temperature (°C); uo, eastwards sea water velocity (m s^−1^) and vo, northwards sea water velocity (m s^−1^).

**Figure 5:**
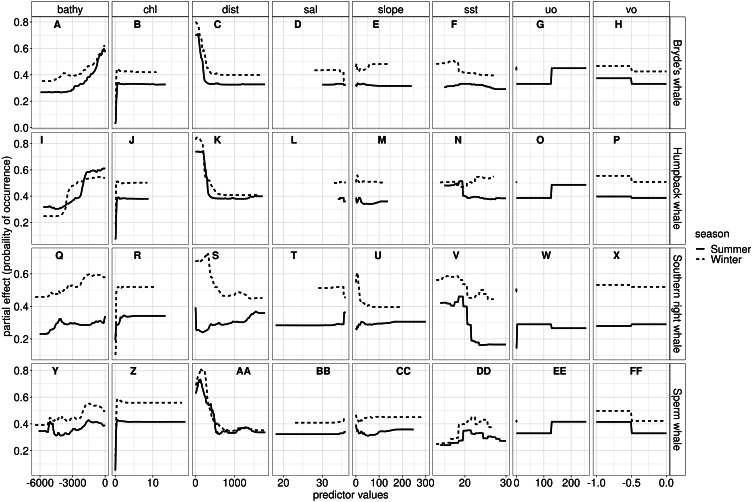
The most influential variables from the RF algorithm for Bryde’s whale, humpback whale, southern right whale and sperm whale during summer and winter. The *y*-axis indicates the probability of occurrence (partial effect), and the *x*-axis indicates the range of the predictor variables. Bathy, bathymetry (m); Chl, chlorophyll-a concentration (mg m^−3^); Dist, distance to shore (km); Sal, salinity (psu); Slope, angle of slope (degrees); SST, sea surface temperature (°C); uo, eastwards sea water velocity (m s ^−1^) and vo, northwards sea water velocity (m s^−1^).

**Figure 6:**
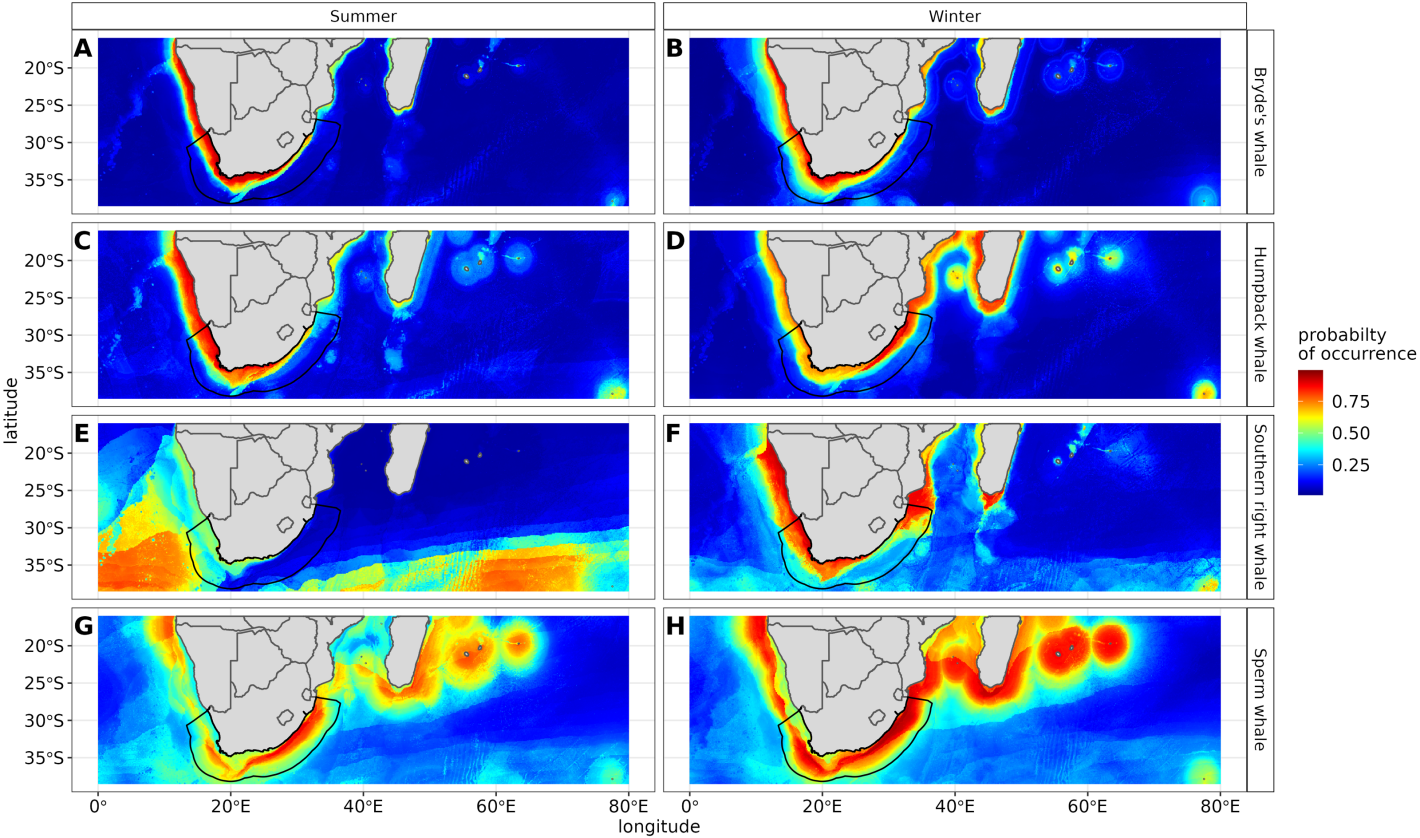
Ensemble model projection using the mean probabilities for each season for the Bryde’s whale, humpback whale, southern right whale and sperm whale. The legend depicts the habitat suitability, the darker red the colour, the higher the predicted occurrence. The South African exclusive economic zone is denoted by the black line.

**Figure 7:**
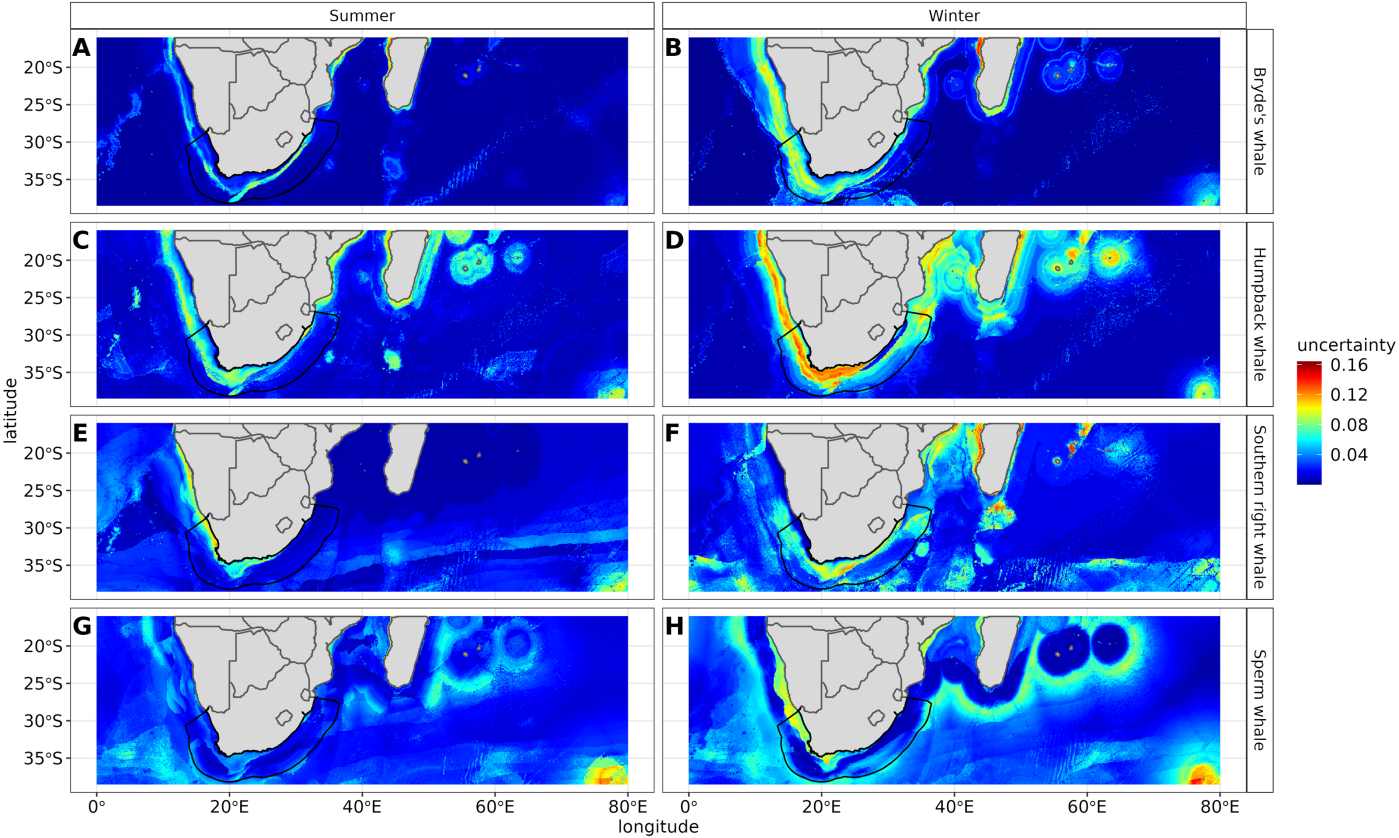
Uncertainty maps measuring algorithm agreement in predictions and depicting regions of varying agreement in the seven different algorithms for Bryde’s whale, humpback whale, southern right whale and sperm whale in both seasons. The higher the uncertainty the more red the colour. The South African exclusive economic zone is denoted by the black line.

The most influential environmental variables driving distribution differed among species, seasons and algorithms (Fig. 4). Figure 5 shows the most influential environmental variables for the RF algorithm, and it indicates predicted preferred ranges for each species (see Figs. S3A–S3F for the most influential environmental variables for the other algorithms). Environmental variables influenced the predicted occurrence of the four cetacean species and can be seen in ensemble model predictions in Fig. 6. Seasonally, southern right whales, sperm whales and humpback whales showed a more pronounced variation in distribution than Bryde’s whales (Fig. 6). Variance in the uncertainty maps for all species during both seasons was low, with values less than 0.16, indicating close agreement between algorithms in the ensemble models (Fig. 7). When looking at the individual algorithms and their predictive ability, the RF algorithm produced probability maps that were more similar to the ensemble model predictions than the other individual algorithms did (Figs. S4A–S4H).

#### Bryde’s whales

SST, distance to shore, chl-a and bathymetry best explained the predicted occurrence of Bryde’s whales during both seasons in the ensemble model, CTA, GBM, MARS and RF algorithms (Fig. 4). According to the RF algorithm, Bryde’s whales were predicted to occur in chl-a concentrations of more than 0.5 mg m^−3^ in summer and more than 0.4 mg m^−3^ in winter (Fig. 5). They were more likely to occur in areas where distance to shore was less than 180 km in summer and less than 100 km in winter, in water depths of less than 700 m in summer and less than 800 m in winter and in SSTs of less than 26 °C in summer and between 16 °C and 25 °C in winter (Fig. 5).

The predictive habitat distribution maps indicate that Bryde’s whales occur in both seasons on the south coast of South Africa, along the west coast of South Africa and along the Namibian coast, remaining in areas that are productive and shallow (Fig. 6). There was a high probability of Bryde’s whales in local distributions along the east coast of South Africa and Mozambique (Fig. 6). The southernmost tip of Madagascar showed a high-predicted prevalence of Bryde’s whales throughout the year. On the west coast of southern Africa, the probability of occurrence increases slightly in the areas offshore in winter (Fig. 6).

#### Humpback whales

In both seasons, distance to shore, chl-a and bathymetry best explained the distribution of humpback whales for the ensemble model, CTA, GBM and RF algorithms (Fig. 4). The RF algorithm indicated that humpback whales are more likely to occur in waters that are less than 200 km from shore during summer and less than 170 km from shore during winter (Fig. 5). They are predicted to occur in waters with chl-a concentrations of less than 4 mg m^−3^ in summer and less than 0.3 mg m^−3^ in winter and water depths of less than 2,000 m in summer and less than 2,500 m in winter (Fig. 5).

The predicted distribution maps show that humpback whales occur slightly further offshore in the summer months, but with a lower probability of occurrence (Fig. 6). The predicted habitat distribution maps indicate that, during winter, humpback whale distribution shrinks to a small coastal band around Madagascar, South Africa and Mozambique with a high probability of occurrence (Fig. 6). Around the west coast of South Africa and Namibia during winter the probability of occurrence is less than the eastern part of southern Africa and Madagascar. This pattern is reversed during summer, where the likelihood of occurrence is greater along the west and south coasts of southern Africa (Fig. 6).

#### Southern right whales

In summer, SST explained the southern right whale predicted occurrence best in the ensemble model as well as all the other algorithms, except ANN (Fig. 4). For the RF algorithm in summer, SST explained nearly 50% of the variation in distribution (Fig. 4) with southern right whales preferring temperatures of less than 20 °C (Fig. 5). The data in Fig. 6 indicates movement towards higher latitudes into cooler waters. The predicted occurrence includes the Agulhas Bank, most of the inshore west coast of southern Africa and offshore of the west coast. Predicted occurrence of southern right whales in the Indian Ocean only occurred south of 30 °S (Fig. 6).

In winter, SST, chl-a and bathymetry explained the predicted distribution for the southern right whale in the ensemble model which was most similar to the MARS algorithm (Fig. 4). For the best performing algorithm, RF, chl-a explained over 40% of the variation in distribution (Fig. 4) with southern right whales occurring in concentrations of more than 0.5 mg m^−3^ (Fig. 5). The predictive habitat distribution maps show that southern right whales are more concentrated around the coastline in winter, ranging from northern Namibia to the southernmost tip of Madagascar (Fig. 6).

#### Sperm whales

The distance to shore in both seasons explained 50% of the sperm whale predicted distribution when looking at the ensemble model. It also dominated all other algorithms (Fig. 4). For the best performing algorithm, RF, sperm whales were predicted to occur at distances of less than 150 km to shore in summer and less than 270 km to shore in winter (Fig. 5).

Sperm whales were predicted to occur throughout the region. They have low probabilities of predicted occurrence in the Mozambique Channel in summer, and for both seasons in the area between 20°S to 30°S and 60°E to 80°E. During winter, the probability of occurrence is higher along the west coast, the east coast and around Madagascar and the Indian Ocean islands (Fig. 6).

## Discussion

### Model performance and sensitivity

The quality of data used in this study varies as the data are obtained from both opportunistic platforms and dedicated scientific surveys. Associated effort, which determines absence data, was not recorded in the opportunistic datasets which, despite its constraints, can be useful for cetacean modelling (Torres et al., 2013). Associated effort provides a comprehensive set of absence data over space and time, enabling more robust models to be built. The selection of pseudo-absences (in the lack of absence data) plays a pivotal role in the outcome of the predicted species distribution. By selecting different numbers of pseudo-absence points based on the recommendations of Barbet-Massin et al. (2012) for each algorithm, this study, along with spatial thinning, was able to produce ensemble models using high performing SDMs.

Certain data in this study, due to their opportunistic nature, were spatially biased (Fig. 1). Spatial thinning was used to reduce this bias, but the predicted occurrence (Fig. 6) showed strong evidence that it had not been eliminated completely. When interpreting the results of this study, readers need to be aware of this and take biases into account when applying this study for spatial planning, management and conservation.

Despite its varied data and the biases, this study produced high performing models (Fig. 3), although the ensemble models did not perform as well as expected. Our study, and a study done by Hao et al. (2020), indicates that ensemble models do not always outperform single algorithms as they produce varied results. Hao et al. (2020) suggest that ensemble models can be outperformed by single algorithms when predicting to distant areas and when single algorithms are finely tuned. In this study, the RF algorithms, on average, produced the highest AUC scores (Table S2). RF algorithms in this study have complex trees with no spatial blocking during validation and are, therefore, prone to overfitting (Hao et al., 2020). Looking at Fig. 5, the response curves of the RF algorithms indicate that overfitting was not substantial, especially when compared to the response curves of the GBM algorithms (Fig. S3C). The response curves of the GBM algorithms are flat, indicating overfitting (Derville et al., 2018). One way to address this would be to use spatial blocking (Hao et al., 2020). In spatial blocking, data are split into different blocks used for either calibrating or validating models, allowing the testing data to be spatially distant from the training data, making it more independent (Hao et al., 2020).

### Key findings

#### Bryde’s whales

Recent genetic work by Penry et al. (2018) suggests that, in South African waters, there are two forms of *B. brydei* consisting of the inshore and offshore stocks. They recommend that the *B. brydei* forms should be separated entirely from *B. edeni* as they appear to be morphologically and genetically different in South African waters. A third stock of Bryde’s whales has been described in the waters of Madagascar (Best, 2001). The recent identification of Omuras whales (*Balaenoptera omurai*) a whale similar to the Bryde’s whale, in the north west of Madagascar (Cerchio et al., 2015), casts some uncertainty on the distribution of Bryde’s whales in Malagasy waters. Due to the lack of clarification of the genetic structure of these whale populations, determining population status from historical whaling records is problematic. These problems are compounded by the whaling records of Bryde’s whale in which there are multiple misidentifications of Bryde’s whales as sei whales (*Balaenoptera borealis*) (Jefferson, Leatherwood & Webber, 1993). Our distribution results confirm Best’s (2001) description of the presence of three stocks of Bryde’s whales in southern Africa. These results must be interpreted with care as they more than likely consist of the two forms of *B brydei, B. edeni* and Omura’s whale.

Little is known about Bryde’s whales’ migration, but Best (2001) indicated that the offshore stock migrates north in the southeastern Atlantic during the spring and summer months, while the inshore stock is resident throughout the year. Our results do not indicate any seasonal variation in habitat preference for the offshore stock. The Agulhas Bank and the west coast are productive systems and vital habitats for pelagic fish that form the diet of the inshore whales (Krug, Tournadre & Dufois, 2014). Our results confirm Best’s (2001) hypothesis that there is little seasonal migration in the inshore whale stocks, presumably owing to the presence of food all year round. In future, separate modelling of the three stocks of Bryde’s whales may provide more information on seasonal distribution.

#### Humpback whales

Humpback whales make seasonal migrations from higher latitudes in summer through the South African EEZ to the coastal waters of more northerly countries (Mozambique, Madagascar, Tanzania, Angola and Gabon) in winter where the water is warmer to calve (Findlay et al., 2011). When their winter breeding season is over, they migrate back to the higher latitudes to feed during the austral summer (Findlay et al., 2011). There is also evidence of the year-round presence of humpback whales in Antarctica (Van Opzeeland et al., 2013) and South Africa (e.g., Findlay & Best, 1995; Barendse et al., 2011; Findlay et al., 2017). Acoustic recordings off the South African west coast indicate that humpback whales are seasonally present (mainly in winter/spring) (Shabangu, 2018). Together, these findings suggest that part of the population migrates seasonally between high and low latitudes, while some individuals do not.

Our data show a slight variation between summer and winter distributions with humpback whales concentrating along the east coast of southern Africa and the Malagasy coastline in winter. Figure 6 shows a strong likelihood of humpback whales close to shore around the west coast and south coast of southern Africa and Namibia in the summer months, which is contrary to what the literature indicates. Humpback whale numbers have increased along the south-west coast of South Africa in the summer months, where there are documented accounts of feeding supergroups in the highly productive waters of the Benguela ecosystem (Findlay & Best, 1995; Findlay et al., 2017). Our results could either indicate the possibility of humpback whales being present all year round or they could be related to sampling bias as there were very few recorded presence points for humpback whales (particularly offshore sightings) in summer. In future, a more extensive database to model humpback whale distribution may provide a model with more sensitive AUC scores and lower prediction in the summer months around the lower latitude coastline.

#### Southern right whales

Southern right whales, like the humpback whales, migrate to lower latitudes in southern Africa to calve during the austral winter (Elwen & Best, 2004). They generally do not migrate further north into warmer waters, although historical records indicate that they were found on the west coast as far north as the southern region of Angola and on the east coast up to Maputo Bay in Mozambique (Best, 2007). Our predicted distribution suggests that their likelihood of occurrence in this region is high (Fig. 6). Heavy harvesting from 1770 onwards resulted in their global numbers declining from between 55,000 and 70,000 to as few as 300 (Best, 2007). Information on their distribution has shown that the species breeding range has shrunk to the region between Port Elizabeth and Cape Town (Best, 2007).

During the summer season, southern right whales move offshore into the south east Atlantic and south to higher latitudes where waters are more productive (Tormosov et al., 1998; Torres et al., 2013). Our data suggest that SST over 20 °C in summer and winter are limiting for southern right whale distributions due to their preference for cooler water temperatures (Mate et al., 2010).

#### Sperm whales

After large scale exploitation was terminated, the sperm whale population was estimated to be 32% of its original numbers (Whitehead, 2002). Recovery is difficult to estimate due to the widespread distribution of the species, ranging in deep waters (>200 m) from the pack ice (males only) to the equator (Jaquet & Whitehead, 1996) with high concentrations between 40°N and 40°S (Best, 2007).

Results from this study suggest that, in southern African waters, sperm whales are widespread, with a low probability of occurrence along the coastal shelf (Fig. 6). Historic whaling records indicate that some males were caught in South African waters between 200 m and 500 m deep, with the majority in waters 2500 m deep. Females, however, were rarely caught in waters less than 1,000 m deep (Best, 1999).

Whaling catch and sighting records indicate that there is a pronounced seasonal distribution of sperm whales that differs by sex and age (e.g., Best, 1999; Findlay & Best, 2016). Acoustic occurrences of sperm whales off the west coast of South Africa also indicate few sperm whale clicks in autumn and winter (Shabangu & Andrew, 2020, unpublished data). Our results show only a slight seasonal difference, with the probability of predicted distribution decreasing in winter. A better understanding of sperm whale distribution and migratory patterns may be attained by modelling separate sexes and age classes and may help improve the understanding of the environmental variables that drive their distribution.

### Management implications

This study shows that southern Africa provides important habitats for these four cetacean species and, according to the literature, fulfil requirements during different life stages in both coastal and offshore habitats throughout the year (Best, 2007). The importance of these areas underpins their need for protection from increasing anthropogenic activities.

Golden et al. (2017) highlight the plight of ocean decision makers in the 21st century: maintaining and expanding an ocean economy to supply the demands of the rapidly growing world population while balancing resource protection through ocean governance. South Africa is not unique in this regard and is currently trying to maintain this balance in the expansion of its ocean economy (Findlay, 2018; Van Wyk, 2015) through the recently implemented MPAs (Findlay, 2020). At a 90% probability of occurrence, the MPA network in South Africa protects less than 10% of seven out of the nine odontocete species modelled habitats (Purdon et al., 2020a; Purdon et al., 2020b). Our results indicate that the predicted occurrence for the four cetaceans studied in this paper is widespread in South Africa, with a small percentage of their habitat being protected by the current MPAs. Important marine mammal areas (IMMAs) could help in identifying more areas that could be delineated as MPAs (Corrigan et al., 2014) in the future.

IMMAs do not have the same regulatory jurisdiction as MPAs over the protection of marine fauna, but they can aid in marine spatial planning, conservation and management of the oceans (Agardy et al., 2019). Delineation of IMMAs is based on four categories, (1) species status based on the International Union for the Conservation of Nature red list, (2) marine mammal distribution and abundance, (3) critical life stage habitats for behaviours such as breeding, feeding and migrating and (4) unique characteristics such as species richness and diversity (Agardy et al., 2019). Recently these criteria were used to identify two IMMAs in South Africa. The first one stretches along the 200 m isobath up the east coast of South Africa and Mozambique. This IMMA was proclaimed to identify the calving areas of humpback whales (Marine Mammal Protected Areas Task Force, 2019a). The second IMMA covers the southern coastal and shelf waters of South Africa, identifying the Indian Ocean humpback dolphin (*Sousa plumbea*), Bryde’s whale (*B. edeni*), Indo-Pacific bottlenose dolphin (*Tursiops aduncus*), common dolphin (*Delphinus delphis*) and the Cape fur seal (*Arctocephalus pusillus*) habitats (Marine Mammal Protected Areas Task Force, 2019b). Within these two IMMAs are not only the species that meet the IMMA criteria but others too. For example, within the second IMMA there are the calving areas of the southern right whale on top of the other eight species it was identified for. These IMMAs, however, include mainly coastal species, reducing the chances of more offshore protection when new MPAs are delineated for offshore species like the sperm whale that occurs on the shelf edge and further offshore. With more distribution modelling and better data, including age and sex classes, critical sperm whale habitats could be identified for IMMAs and eventually protected through MPAs.

## Conclusion

This study has shown that the use of multiple databases, spanning various spatial and temporal frames, can give baseline knowledge of the environmental variables that drive species distribution. There are limitations to using this type of dataset, and results should be interpreted with appropriate care. For example, by looking at the Bryde’s whale predicted distribution, it appears as if they are widely spread throughout southern Africa. However, a closer look at their stock structure has indicated that this distribution is possibly split between the two forms of *B brydei, B. edeni* and Omura’s whales. This study also highlights the importance of accounting for biases in the data structure. For example, by spatially thinning the presence data, some but not all spatial biases were removed. Also, to prevent overfitting and improve this study, spatial blocks should be used, similar to the study by Hao et al. (2020). Overall, the use of ensemble models does not necessarily provide the best model performance as individual algorithms can produce AUC values that are as high, or higher than ensemble models. Without *a priori* knowledge of algorithm performance, however, the ensemble models remain a robust option with good generalised performance across species and seasons. Results such as these can be used to identify key areas and potential IMMAs or MPAs that need to be examined in more detail to protect cetaceans from increasing anthropogenic activities.

##  Supplemental Information

10.7717/peerj.9997/supp-1 Supplemental Information 1 Observed presence data (blue) with an example of one out of the ten selected pseudo-absence sets (red) for each algorithm run to create the ensemble model for Bryde’s whale summer

10.7717/peerj.9997/supp-2 Supplemental Information 2 Observed presence data (blue) with an example of one out of the ten selected pseudo-absence sets (red) for each algorithm run to create the ensemble model for Bryde’s whale winter

10.7717/peerj.9997/supp-3 Supplemental Information 3 Observed presence data (blue) with an example of one out of the ten selected pseudo-absence sets (red) for each algorithm run to create the ensemble model for humpback whale summer

10.7717/peerj.9997/supp-4 Supplemental Information 4 Observed presence data (blue) with an example of one out of the ten selected pseudo-absence sets (red) for each algorithm run to create the ensemble model for humpback whale winter

10.7717/peerj.9997/supp-5 Supplemental Information 5 Observed presence data (blue) with an example of one out of the ten selected pseudo-absence sets (red) for each algorithm run to create the ensemble model for southern right whale summer

10.7717/peerj.9997/supp-6 Supplemental Information 6 Observed presence data (blue) with an example of one out of the ten selected pseudo-absence sets (red) for each algorithm run to create the ensemble model for southern right whale winter

10.7717/peerj.9997/supp-7 Supplemental Information 7 Observed presence data (blue) with an example of one out of the ten selected pseudo-absence sets (red) for each algorithm run to create the ensemble model for sperm whale summer

10.7717/peerj.9997/supp-8 Supplemental Information 8 Observed presence data (blue) with an example of one out of the ten selected pseudo-absence sets (red) for each algorithm run to create the ensemble model for sperm whale winter

10.7717/peerj.9997/supp-9 Supplemental Information 9 Summer oceanographic and topographic variables used to create the final ensemble model for the Bryde’s whale, humpback whale, southern right whale and sperm whale Bathy, depth (m); Chl, chlorophyll a concentration (mg m−3); Dist., distance to shore (km); sst, sea surface temperature (°C); slope, angle of slope (degrees), sal, salinity (psu), uo, eastwards sea water velocity (m s^−1^); vo, northwards sea water velocity (m s^−1^). Scale bar for each variable is shown on the top axis.

10.7717/peerj.9997/supp-10 Supplemental Information 10 Winter oceanographic and topographic variables used to create the final ensemble model for the Bryde’s whale, humpback whale, southern right whale and sperm whale Bathy, depth (m); Chl, chlorophyll a concentration (mg m−3); Dist., distance to shore (km); sst, sea surface temperature (°C); slope, angle of slope (degrees), sal, salinity (psu), uo, eastwards sea water velocity (m s^−1^); vo, northwards sea water velocity (m s^−1^). Scale bar for each variable is shown on the top axis.

10.7717/peerj.9997/supp-11 Supplemental Information 11 The most influencial variables from the ANN algorithm for Bryde’s whale, humpback whale, southern right whale and sperm whale during the summer and winter seaons *Y* axis indicates the liklihood of occurrence. The *x*-axis indicates the range of the variables; Bathy, depth (m); Chl, chlorophyll a concentration (mg m−3); Dist., distance to shore (km); sst, sea surface temperature (°C); slope, angle of slope (degrees), sal, salinity (psu), uo, eastwards sea water velocity (m s^−1^); vo, northwards sea water velocity (m s^−1^).

10.7717/peerj.9997/supp-12 Supplemental Information 12 The most influencial variables from the CTA algorithm for Bryde’s whale, humpback whale, southern right whale and sperm whale during the summer and winter seaons *Y* axis indicates the liklihood of occurrence. The *x*-axis indicates the range of the variables; Bathy, depth (m); Chl, chlorophyll a concentration (mg m −3); Dist., distance to shore (km); sst, sea surface temperature (°C); slope, angle of slope (degrees), sal, salinity (psu), uo, eastwards sea water velocity (m s^−1^); vo, northwards sea water velocity (m s^−1^).

10.7717/peerj.9997/supp-13 Supplemental Information 13 The most influencial variables from the GBM algorithm for Bryde’s whale, humpback whale, southern right whale and sperm whale during the summer and winter seaons *Y* axis indicates the liklihood of occurrence. The *x*-axis indicates the range of the variables; Bathy, depth (m); Chl, chlorophyll a concentration (mg m −3); Dist., distance to shore (km); sst, sea surface temperature (°C); slope, angle of slope (degrees); sal, salinity (psu), uo, eastwards sea water velocity (m s^−1^); vo, northwards sea water velocity (m s^−1^).

10.7717/peerj.9997/supp-14 Supplemental Information 14 The most influencial variables from the GLM algorithm for Bryde’s whale, humpback whale, southern right whale and sperm whale during the summer and winter seaons *Y* axis indicates the liklihood of occurrence. The x-axis indicates the range of the variables; Bathy, depth (m); Chl, chlorophyll a concentration (mg m−3); Dist., distance to shore (km); sst, sea surface temperature (°C); slope, angle of slope (degrees); sal, salinity (psu), uo, eastwards sea water velocity (m s^−1^); vo, northwards sea water velocity (m s^−1^).

10.7717/peerj.9997/supp-15 Supplemental Information 15 The most influencial variables from the MARS algorithm for Bryde’s whale, humpback whale, southern right whale and sperm whale during the summer and winter seaons Y axis indicates the liklihood of occurrence. The x-axis indicates the range of the variables; Bathy, depth (m); Chl, chlorophyll a concentration (mg m −3); Dist., distance to shore (km); sst, sea surface temperature (°C); slope, angle of slope (degrees); sal, salinity (psu); uo, eastwards sea water velocity (m s^−1^); vo, northwards sea water velocity (m s^−1^).

10.7717/peerj.9997/supp-16 Supplemental Information 16 The most influencial variables from the SVM algorithm for Bryde’s whale, humpback whale, southern right whale and sperm whale during the summer and winter seaons Y axis indicates the liklihood of occurrence. The x-axis indicates the range of the variables; Bathy, depth (m); Chl, chlorophyll a concentration (mg m −3); Dist., distance to shore (km); sst, sea surface temperature (°C); slope, angle of slope (degrees); sal, salinity (psu); uo, eastwards sea water velocity (m s^−1^); vo, northwards sea water velocity (m s^−1^).

10.7717/peerj.9997/supp-17 Supplemental Information 17 Predicted probability of occurrence for Bryde’s whale summer. Scale bars on the right of the maps depict the habitat suitability, the darker red the colour, the higher the probability of occurrence

10.7717/peerj.9997/supp-18 Supplemental Information 18 Predicted probability of occurrence for Bryde’s whale winter. Scale bars on the right of the maps depict the habitat suitability, the darker red the colour, the higher the probability of occurrence

10.7717/peerj.9997/supp-19 Supplemental Information 19 Predicted probability of occurrence for humpback whale summer. Scale bars on the right of the maps depict the habitat suitability, the darker red the colour, the higher the probability of occurrence

10.7717/peerj.9997/supp-20 Supplemental Information 20 Predicted probability of occurrence for humpback whale winter. Scale bars on the right of the maps depict the habitat suitability, the darker red the colour, the higher the probability of occurrence

10.7717/peerj.9997/supp-21 Supplemental Information 21 Predicted probability of occurrence for southern right whale summer. Scale bars on the right of the maps depict the habitat suitability, the darker red the colour, the higher the probability of occurrence

10.7717/peerj.9997/supp-22 Supplemental Information 22 Predicted probability of occurrence for southern right whale winter. Scale bars on the right of the maps depict the habitat suitability, the darker red the colour, the higher the probability of occurrence

10.7717/peerj.9997/supp-23 Supplemental Information 23 Predicted probability of occurrence for sperm whale summer. Scale bars on the right of the maps depict the habitat suitability, the darker red the colour, the higher the probability of occurrence

10.7717/peerj.9997/supp-24 Supplemental Information 24 Predicted probability of occurrence for sperm whale winter. Scale bars on the right of the maps depict the habitat suitability, the darker red the colour, the higher the probability of occurrence

10.7717/peerj.9997/supp-25 Table S1 The number of individual sightings for Bryde’s whale, humpback whale, southern right whale and sperm whale from the different platforms used in this study. Boldfaced values are the total number of sightings per platform provider

10.7717/peerj.9997/supp-26 Table S2 AUC scores for each algorithm and ensemble model for Bryde’s whale, humpback whale, southern right whale and sperm whale during summer and winter

10.7717/peerj.9997/supp-27 Supplemental Information 27 Data used to model the distribution of Bryde’s whale, humpback whale, southern right whale and sperm whale

10.7717/peerj.9997/supp-28 Supplemental Information 28 Custom utility functions

10.7717/peerj.9997/supp-29 Supplemental Information 29 Process model

10.7717/peerj.9997/supp-30 Supplemental Information 30 Prepare process

10.7717/peerj.9997/supp-31 Supplemental Information 31 Prepare process of raw data

10.7717/peerj.9997/supp-32 Supplemental Information 32 SSDM distribution modelling

10.7717/peerj.9997/supp-33 Supplemental Information 33 Prepare base map

10.7717/peerj.9997/supp-34 Supplemental Information 34 Prepare tables

## Additional Information and Declarations

### Data Availability

The following information was supplied regarding data availability: Raw data for each species for each season and the R code are available as Supplemental Files.
