# Supplementary figures and images for: Species distribution modelling of Bryde’s whales, humpback whales, southern right whales, and sperm whales in the southern African region to inform their conservation in expanding economies
Source: PeerJ. 2020 Sep 22;8:e9997. doi: 10.7717/peerj.9997 (PMC7518163; doi:10.7717/peerj.9997)

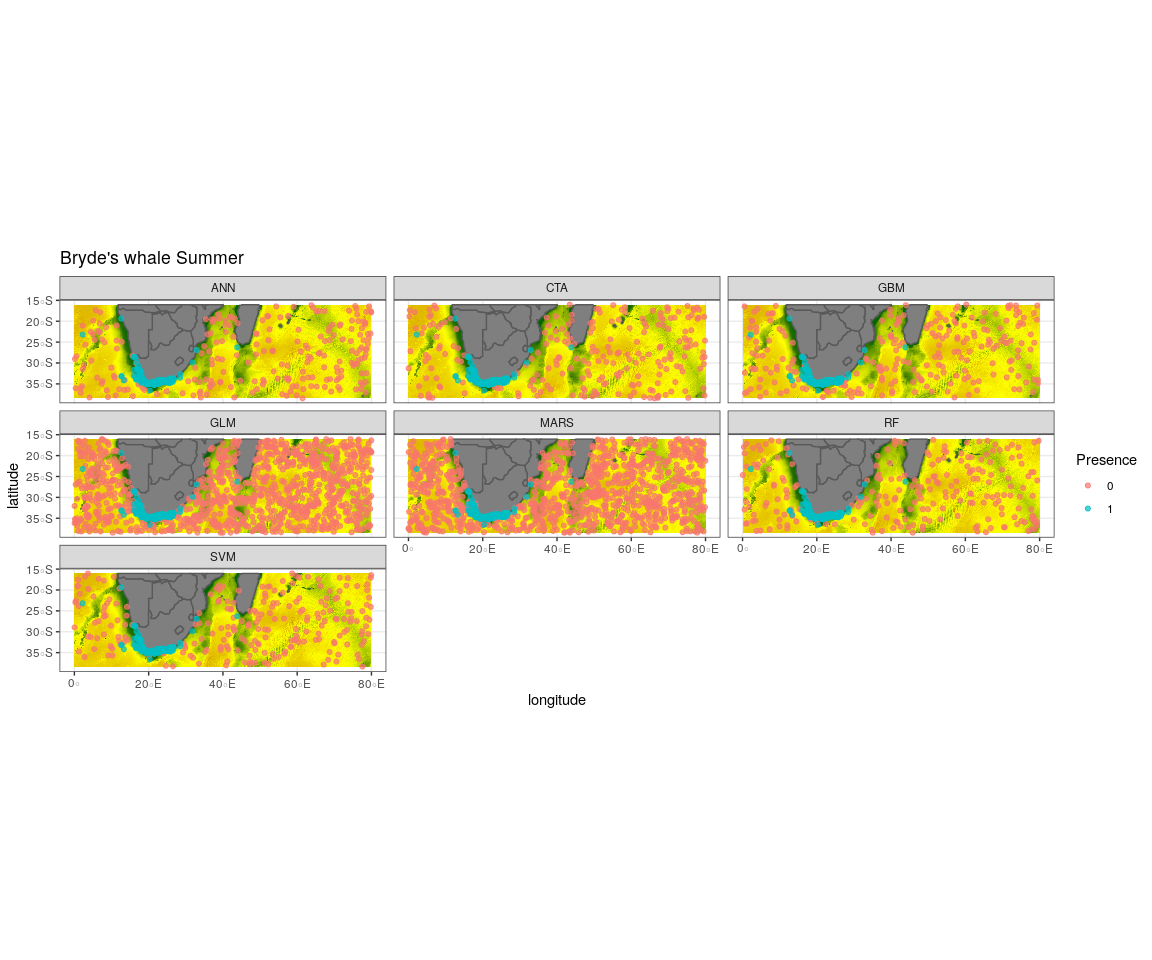

Supplement: Supplemental Information 1 [file peerj-08-9997-s001.png]

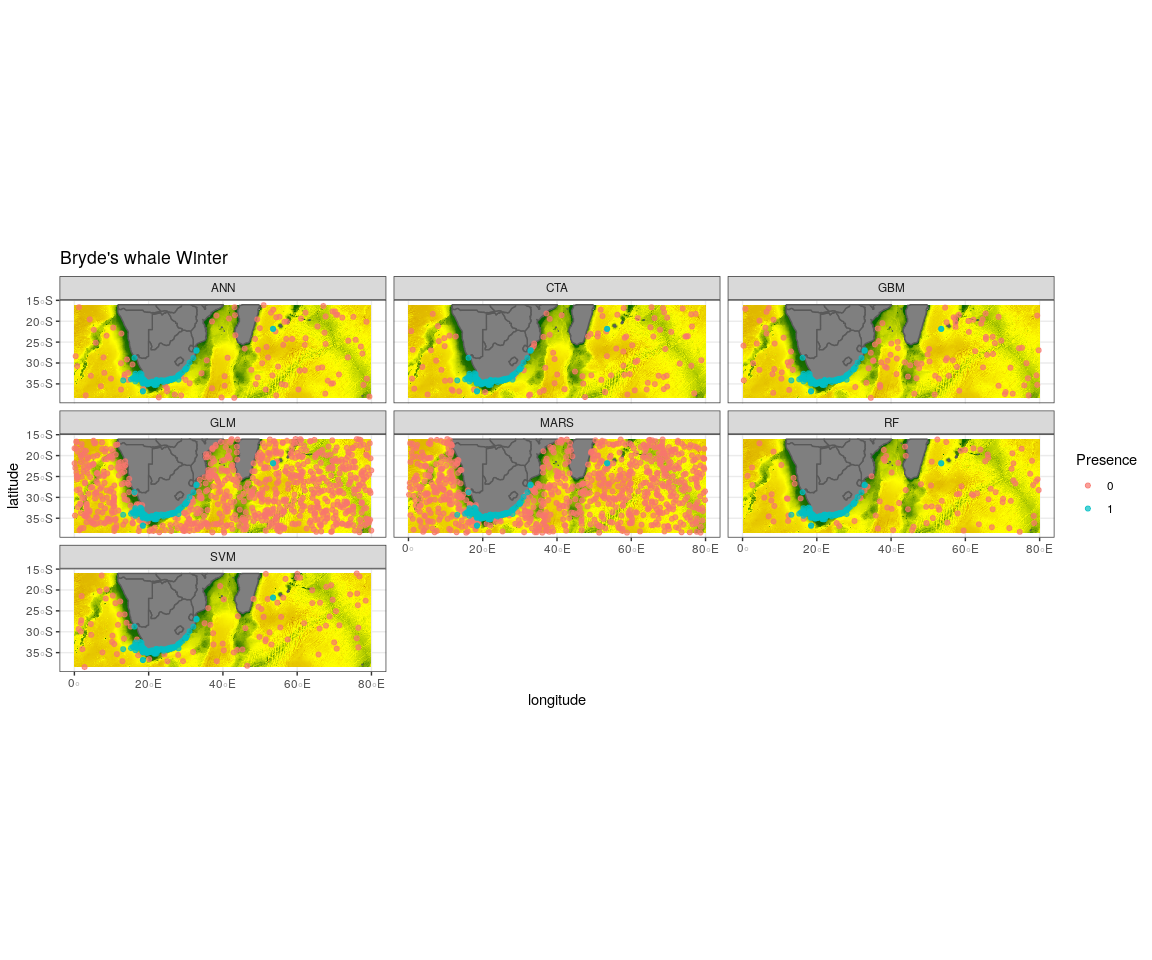

Supplement: Supplemental Information 2 [file peerj-08-9997-s002.png]

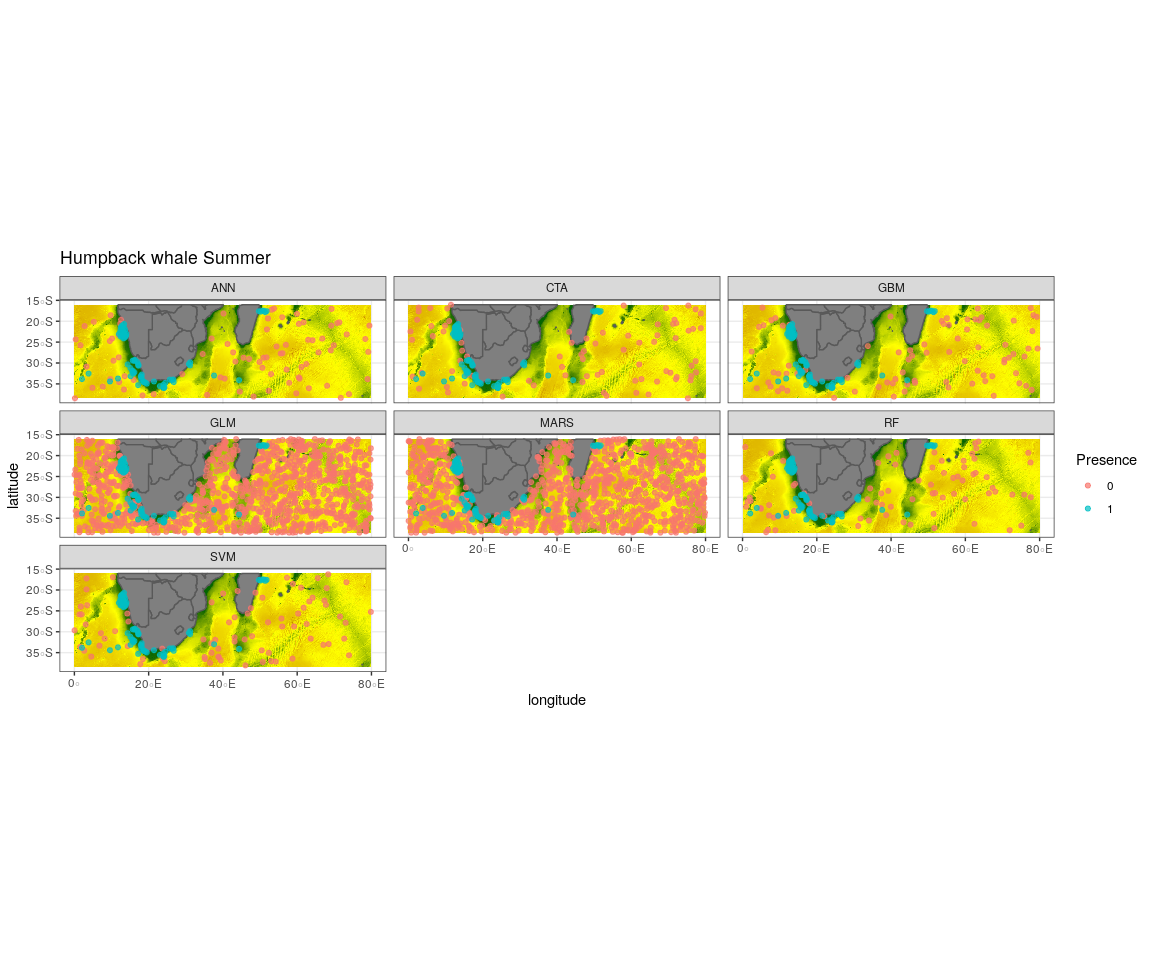

Supplement: Supplemental Information 3 [file peerj-08-9997-s003.png]

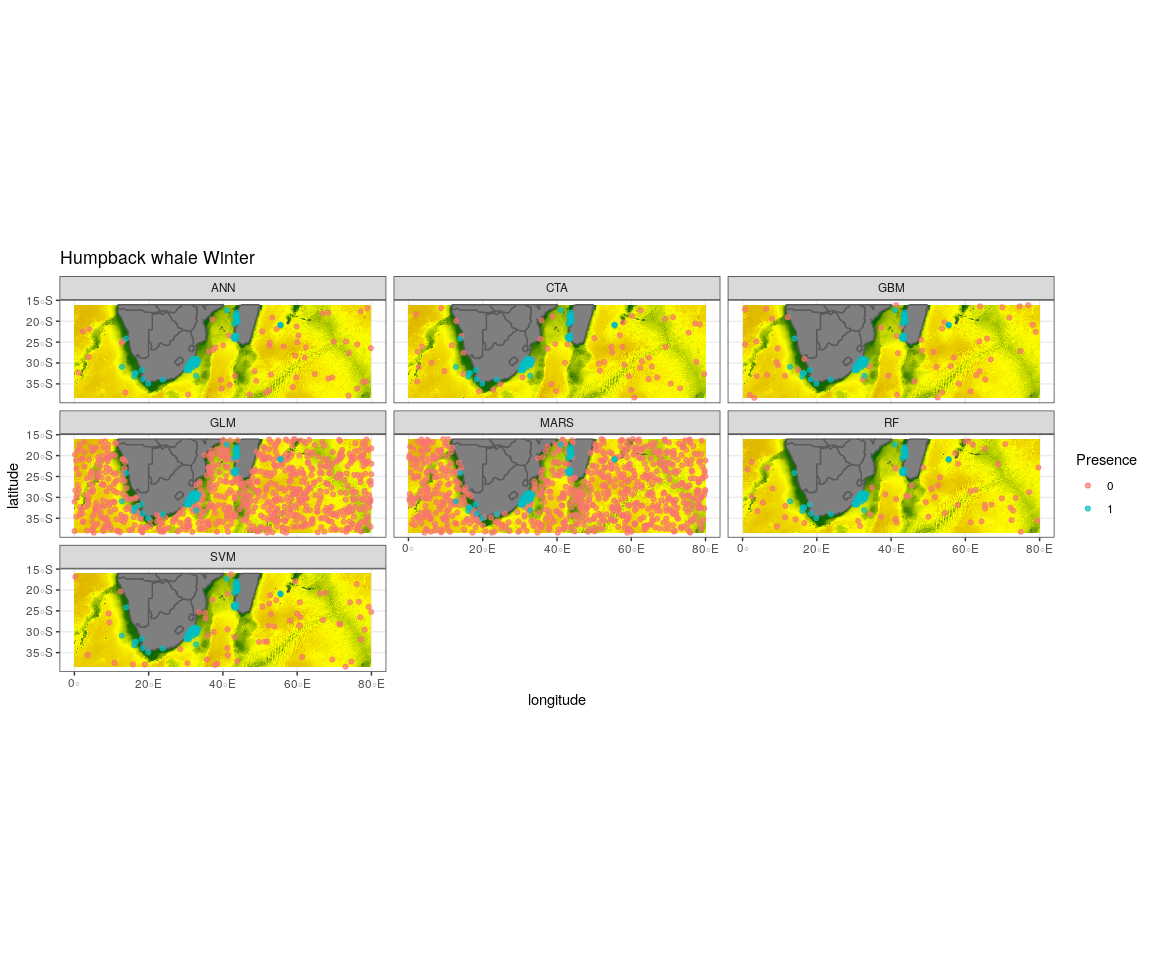

Supplement: Supplemental Information 4 [file peerj-08-9997-s004.png]

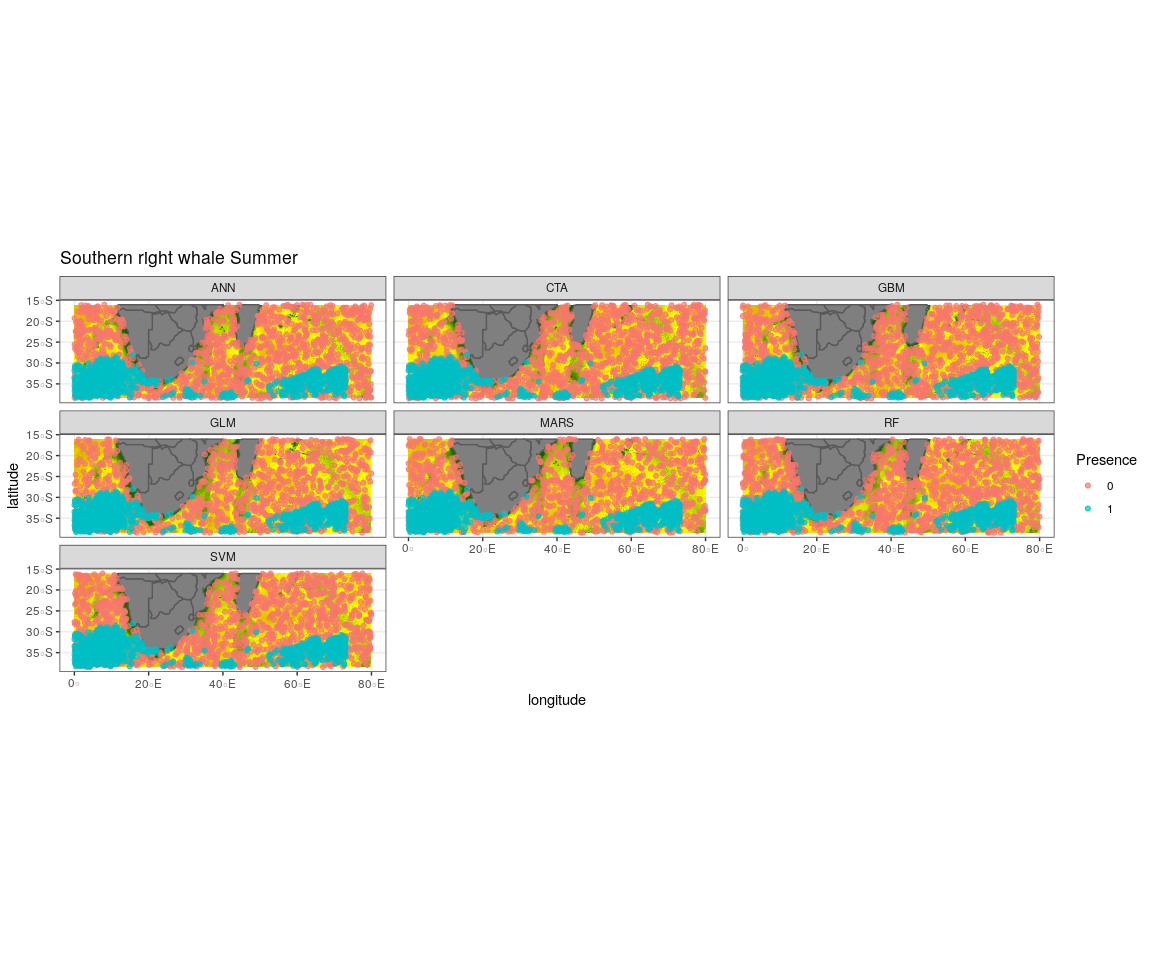

Supplement: Supplemental Information 5 [file peerj-08-9997-s005.png]

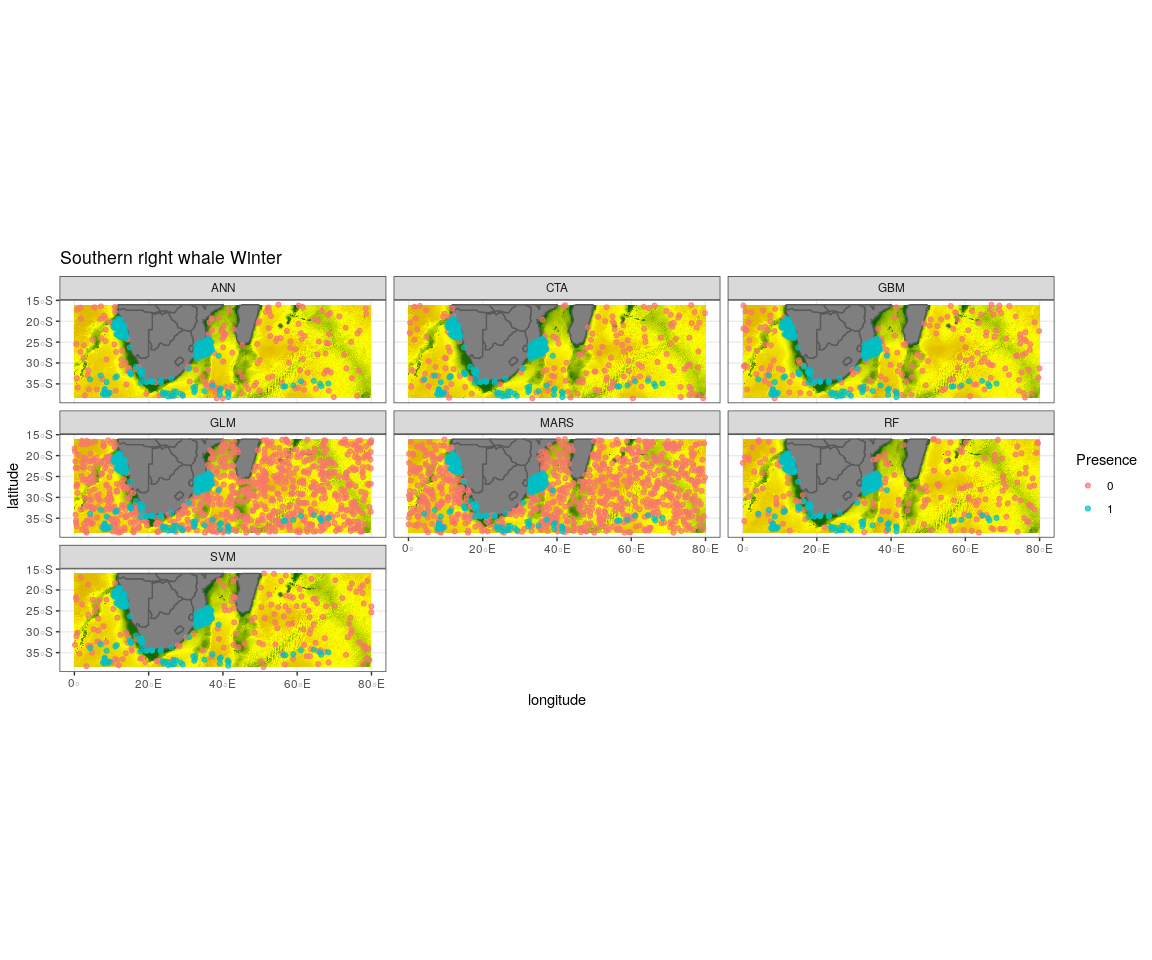

Supplement: Supplemental Information 6 [file peerj-08-9997-s006.png]

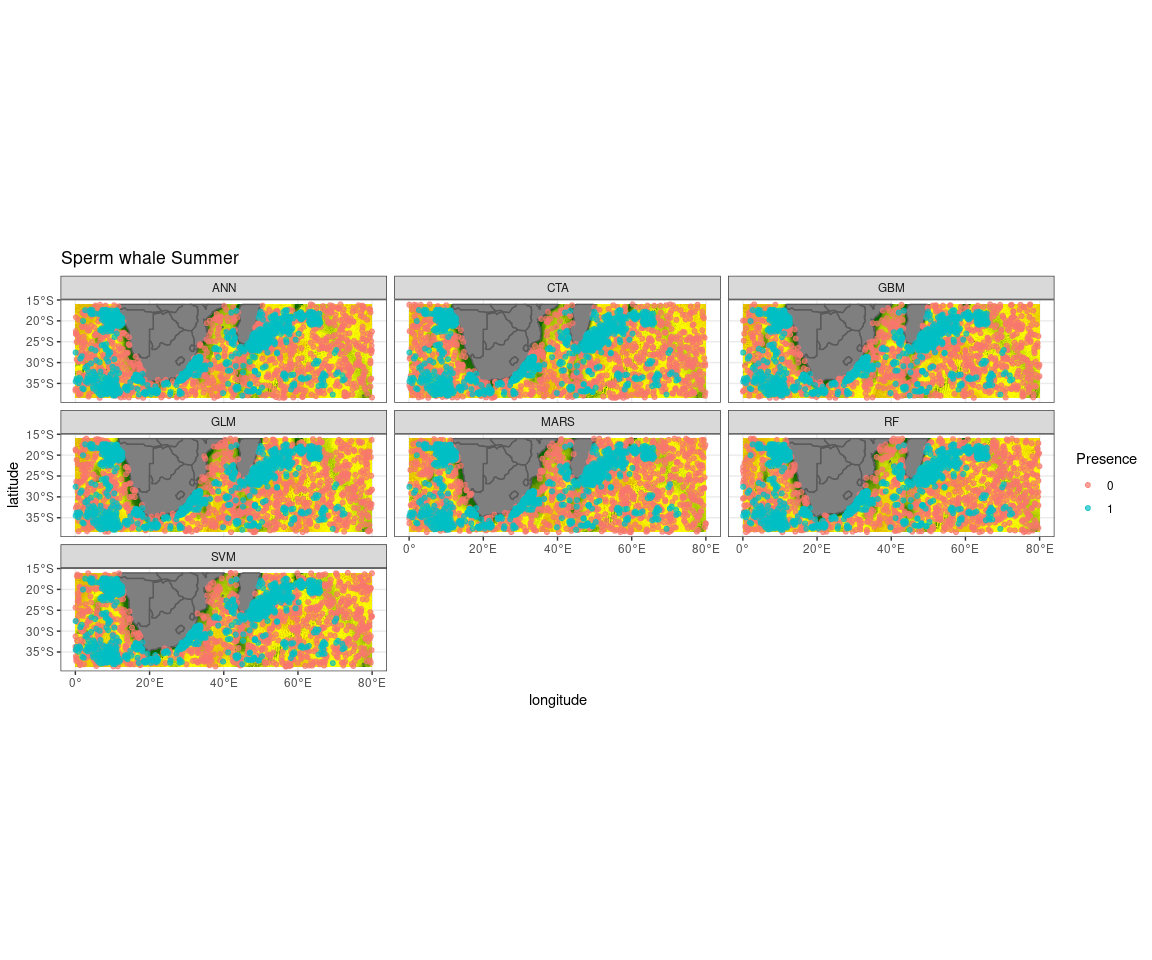

Supplement: Supplemental Information 7 [file peerj-08-9997-s007.png]

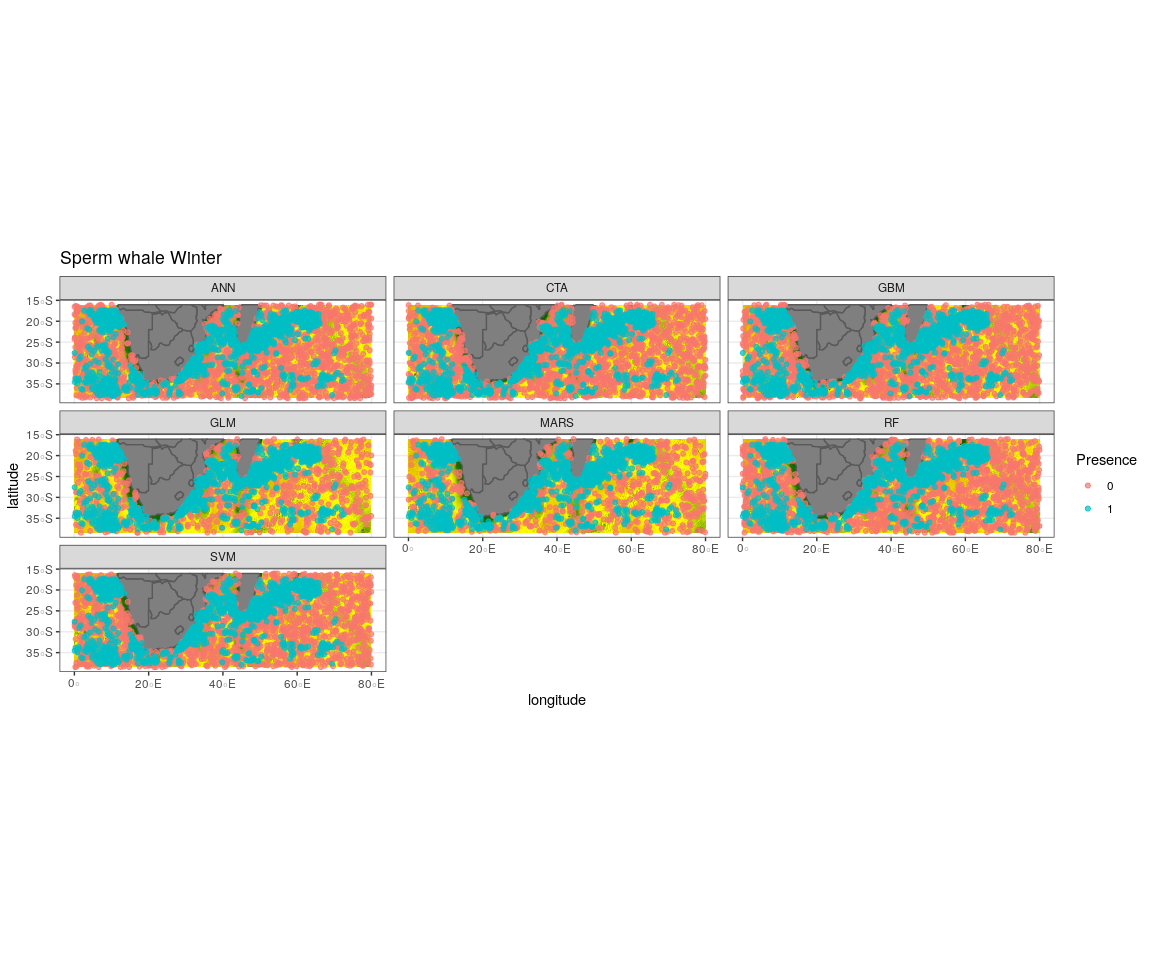

Supplement: Supplemental Information 8 [file peerj-08-9997-s008.png]

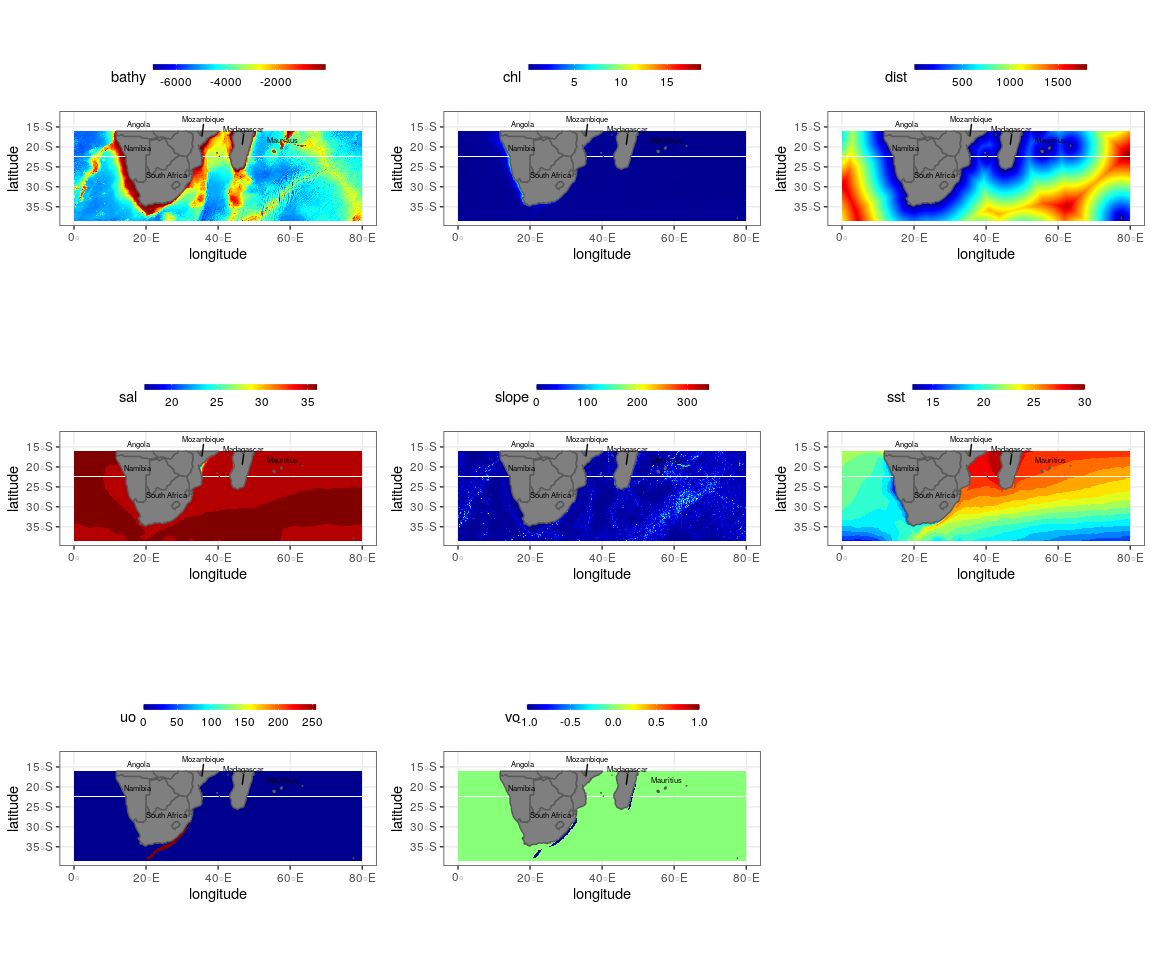

Supplement: Supplemental Information 9 — Bathy, depth (m); Chl, chlorophyll a concentration (mg m−3); Dist., distance to shore (km); sst, sea surface temperature (°C); slope, angle of slope (degrees), sal, salinity (psu), uo, eastwards sea water velocity (m s−1); vo, northwards sea water velocity (m s−1). Scale bar for each variable is shown on the top axis. [file peerj-08-9997-s009.png]

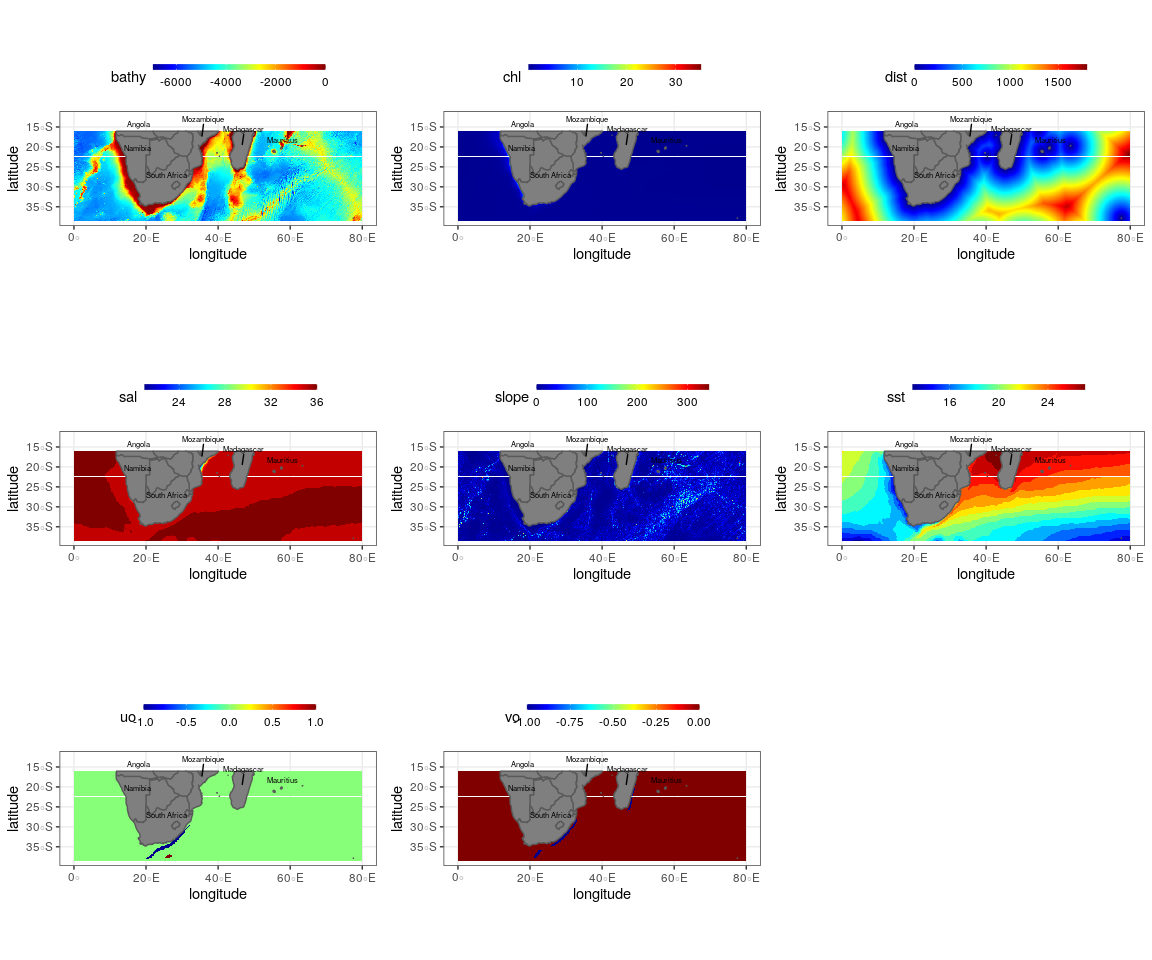

Supplement: Supplemental Information 10 — Bathy, depth (m); Chl, chlorophyll a concentration (mg m−3); Dist., distance to shore (km); sst, sea surface temperature (°C); slope, angle of slope (degrees), sal, salinity (psu), uo, eastwards sea water velocity (m s−1); vo, northwards sea water velocity (m s−1). Scale bar for each variable is shown on the top axis. [file peerj-08-9997-s010.png]

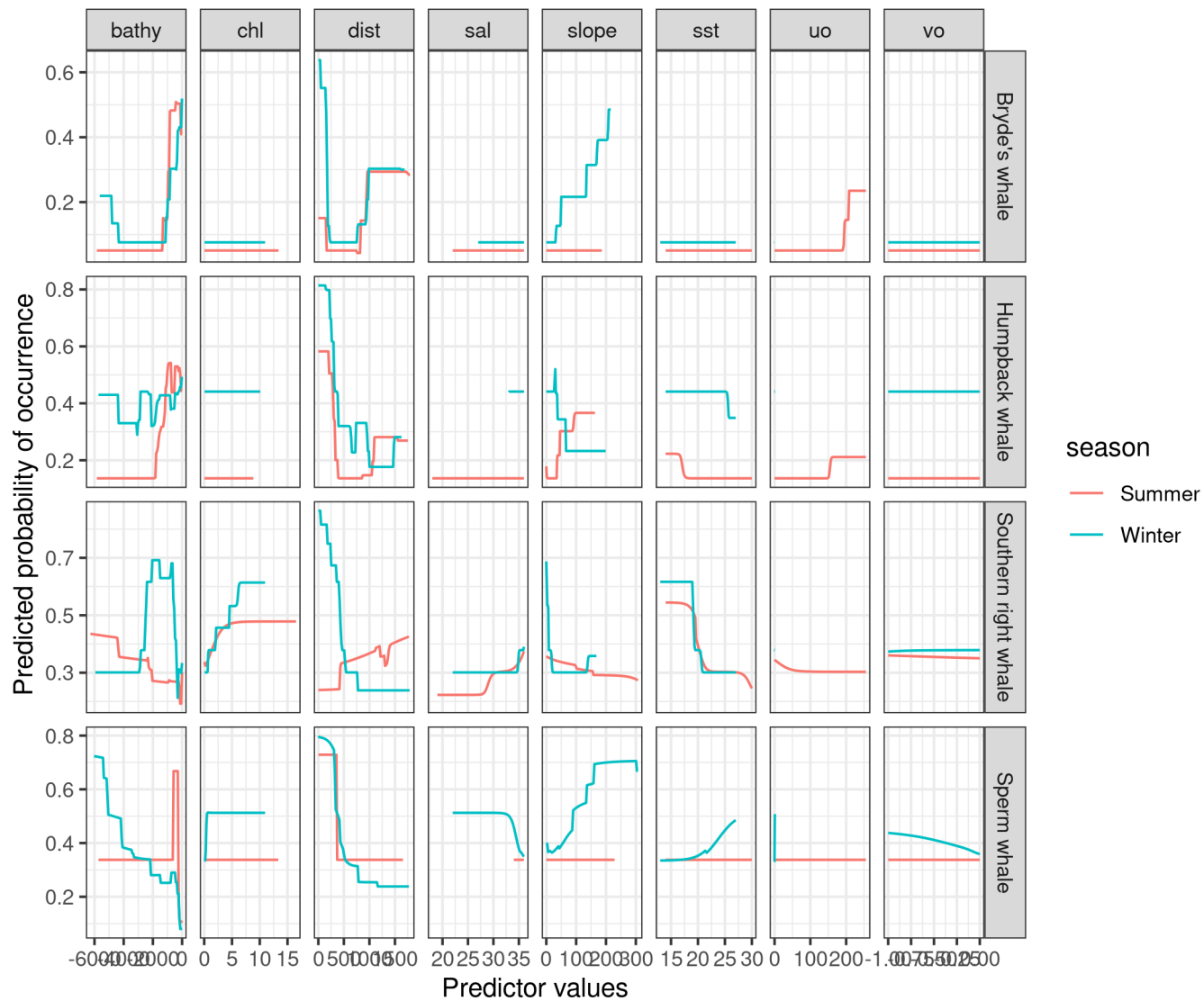

Supplement: Supplemental Information 11 — Y axis indicates the liklihood of occurrence. The x-axis indicates the range of the variables; Bathy, depth (m); Chl, chlorophyll a concentration (mg m−3); Dist., distance to shore (km); sst, sea surface temperature (°C); slope, angle of slope (degrees), sal, salinity (psu), uo, eastwards sea water velocity (m s−1); vo, northwards sea water velocity (m s−1). [file peerj-08-9997-s011.pdf]

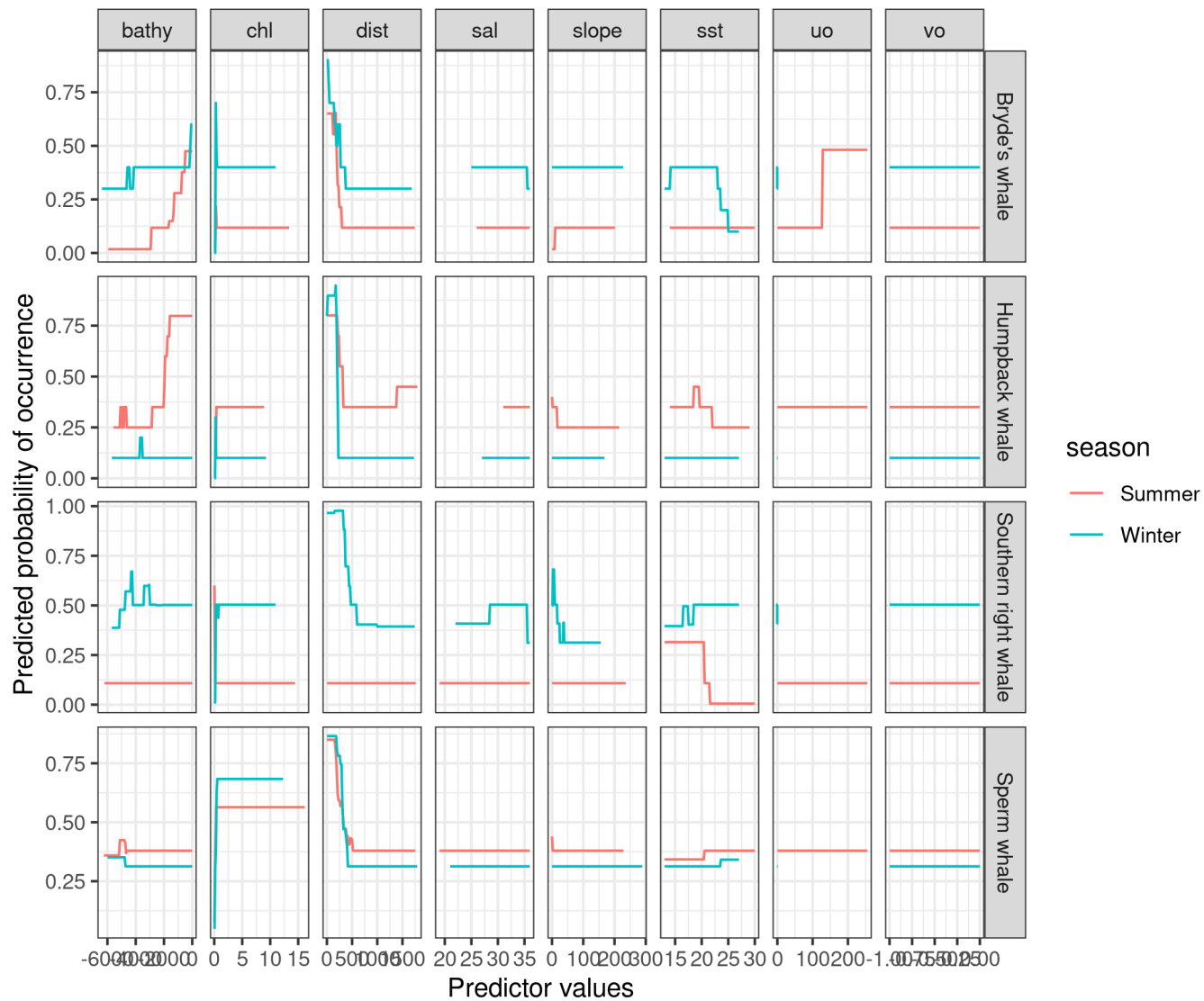

Supplement: Supplemental Information 12 — Y axis indicates the liklihood of occurrence. The x-axis indicates the range of the variables; Bathy, depth (m); Chl, chlorophyll a concentration (mg m −3); Dist., distance to shore (km); sst, sea surface temperature (°C); slope, angle of slope (degrees), sal, salinity (psu), uo, eastwards sea water velocity (m s−1); vo, northwards sea water velocity (m s−1). [file peerj-08-9997-s012.pdf]

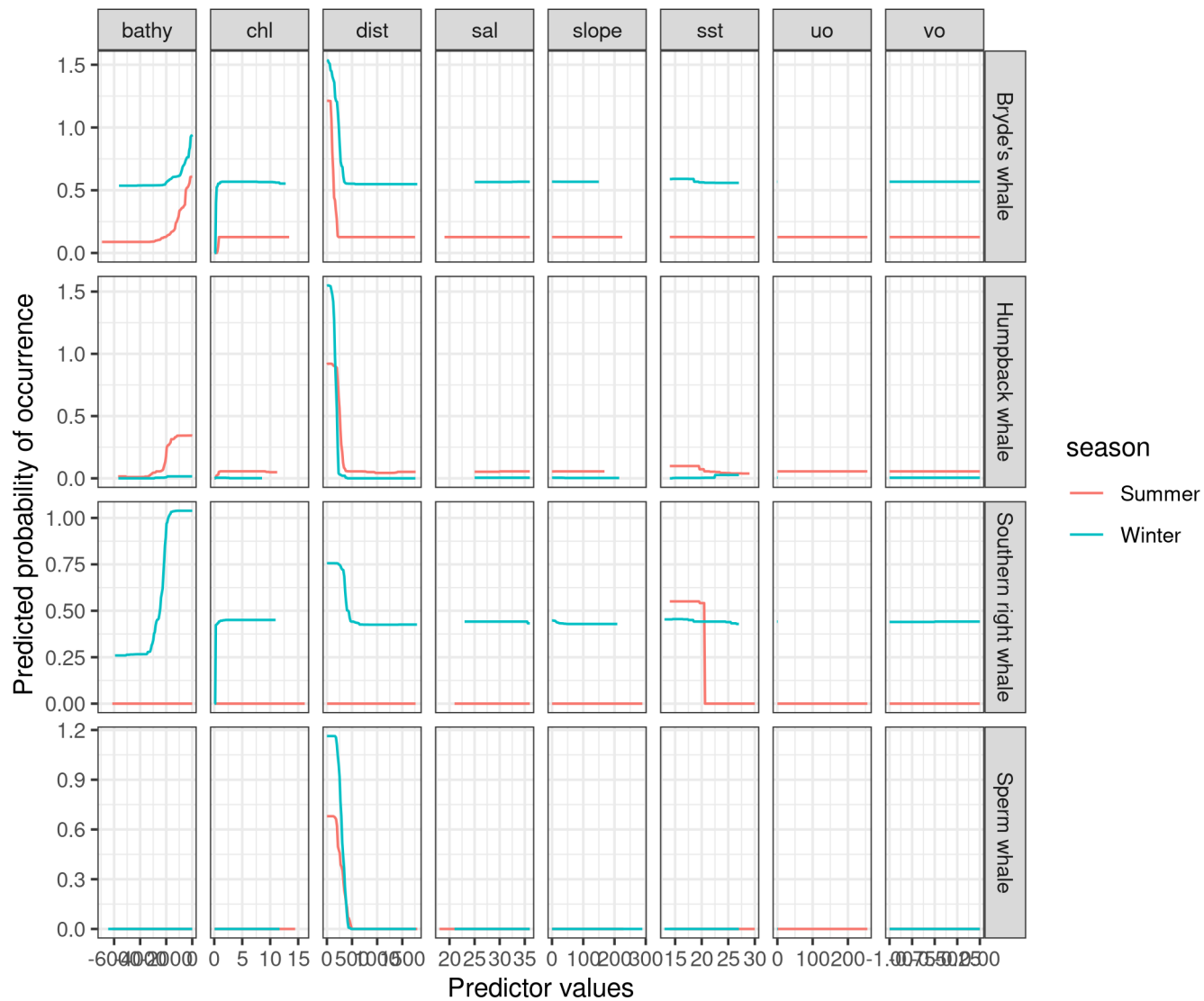

Supplement: Supplemental Information 13 — Y axis indicates the liklihood of occurrence. The x-axis indicates the range of the variables; Bathy, depth (m); Chl, chlorophyll a concentration (mg m −3); Dist., distance to shore (km); sst, sea surface temperature (°C); slope, angle of slope (degrees); sal, salinity (psu), uo, eastwards sea water velocity (m s−1); vo, northwards sea water velocity (m s−1). [file peerj-08-9997-s013.pdf]

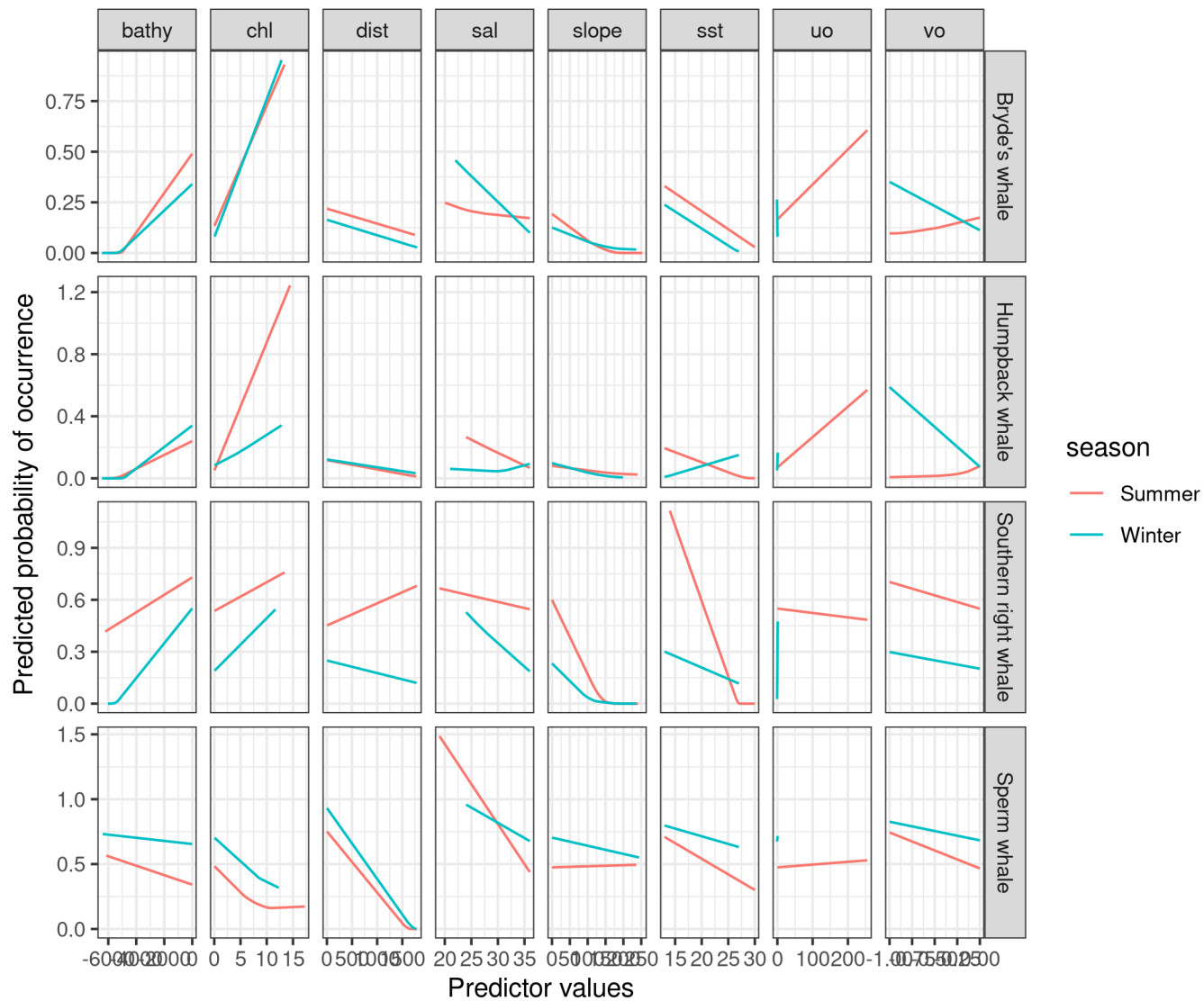

Supplement: Supplemental Information 14 — Y axis indicates the liklihood of occurrence. The x-axis indicates the range of the variables; Bathy, depth (m); Chl, chlorophyll a concentration (mg m−3); Dist., distance to shore (km); sst, sea surface temperature (°C); slope, angle of slope (degrees); sal, salinity (psu), uo, eastwards sea water velocity (m s−1); vo, northwards sea water velocity (m s−1). [file peerj-08-9997-s014.pdf]

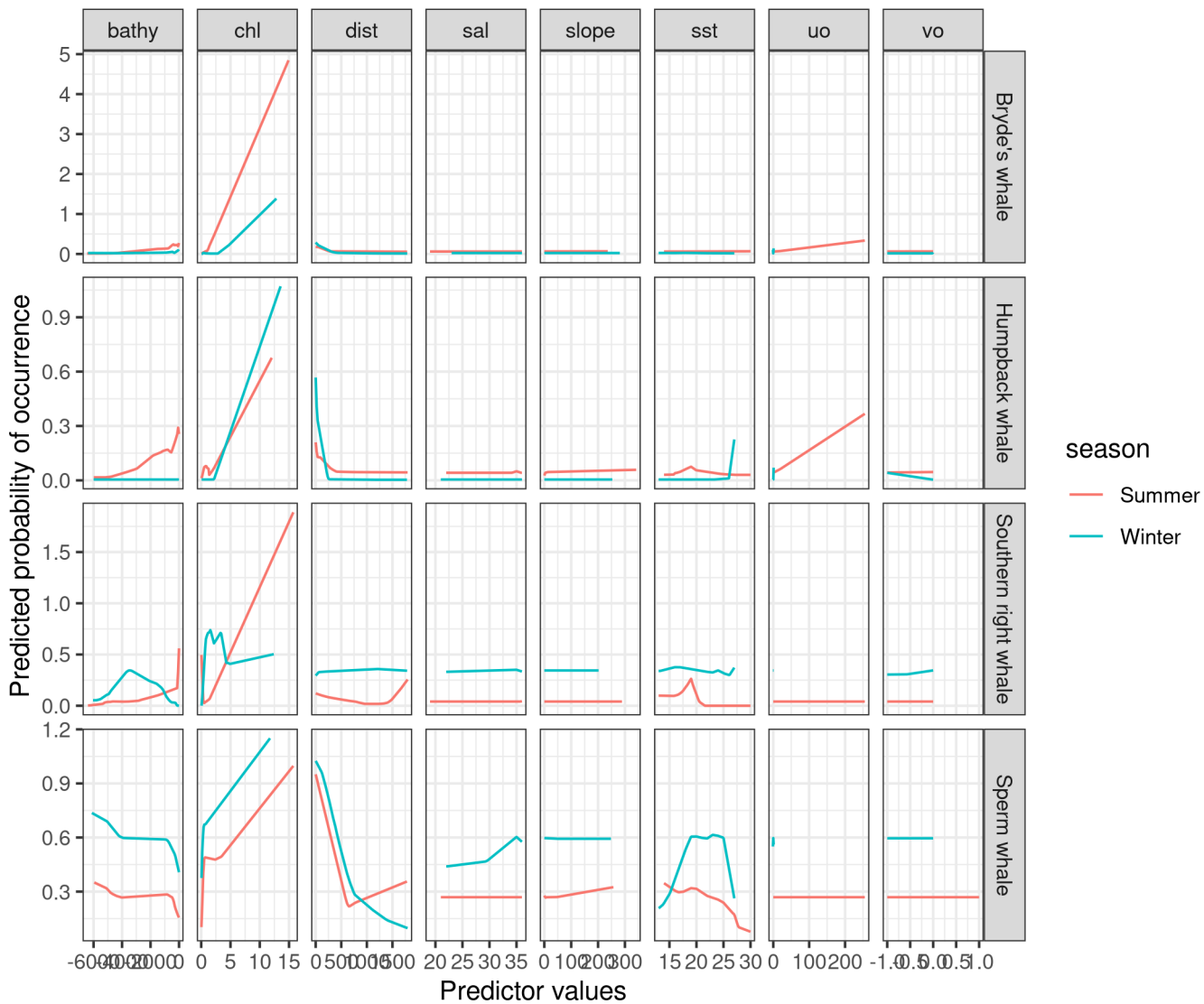

Supplement: Supplemental Information 15 — Y axis indicates the liklihood of occurrence. The x-axis indicates the range of the variables; Bathy, depth (m); Chl, chlorophyll a concentration (mg m −3); Dist., distance to shore (km); sst, sea surface temperature (°C); slope, angle of slope (degrees); sal, salinity (psu); uo, eastwards sea water velocity (m s−1); vo, northwards sea water velocity (m s−1). [file peerj-08-9997-s015.pdf]

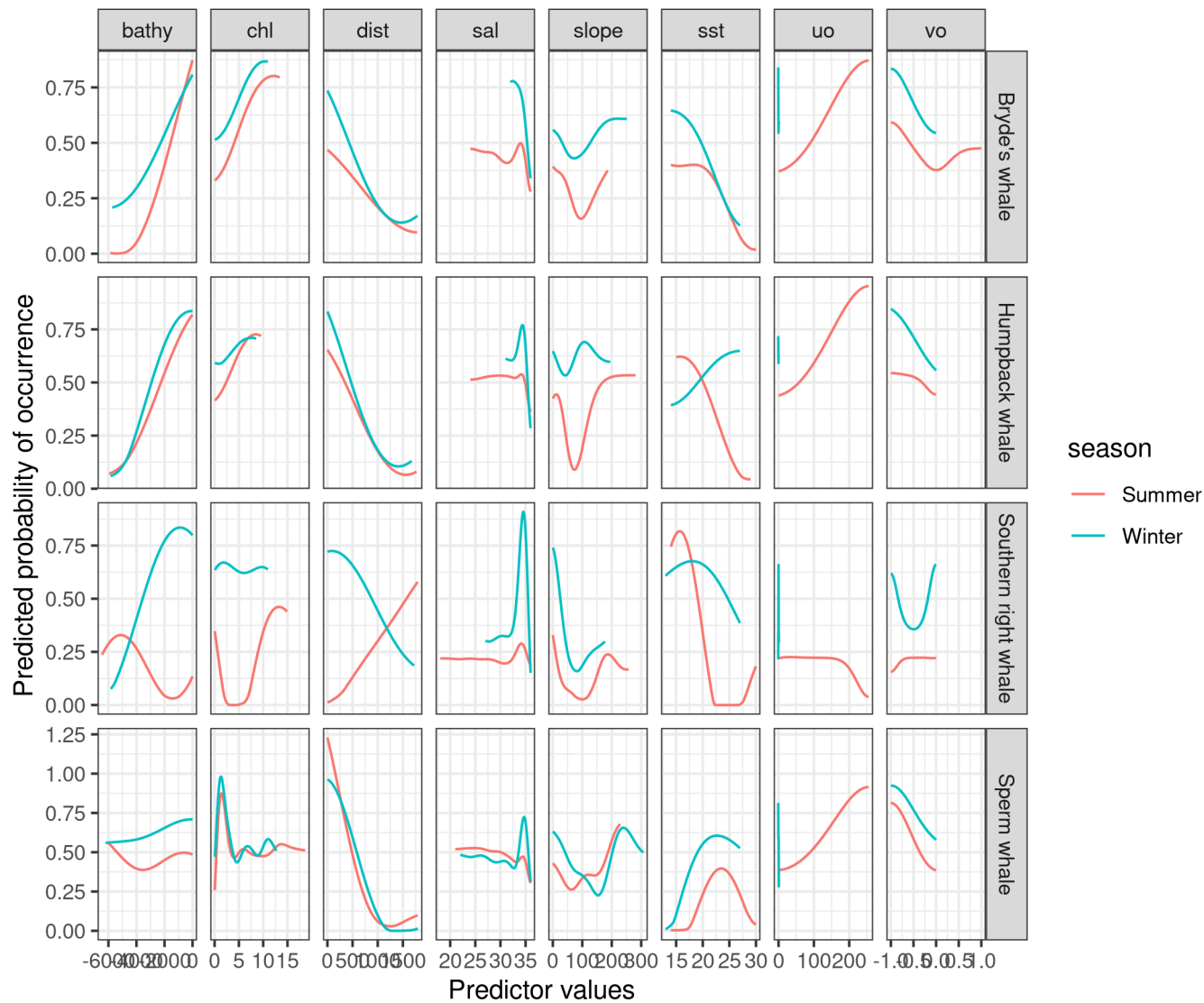

Supplement: Supplemental Information 16 — Y axis indicates the liklihood of occurrence. The x-axis indicates the range of the variables; Bathy, depth (m); Chl, chlorophyll a concentration (mg m −3); Dist., distance to shore (km); sst, sea surface temperature (°C); slope, angle of slope (degrees); sal, salinity (psu); uo, eastwards sea water velocity (m s−1); vo, northwards sea water velocity (m s−1). [file peerj-08-9997-s016.pdf]

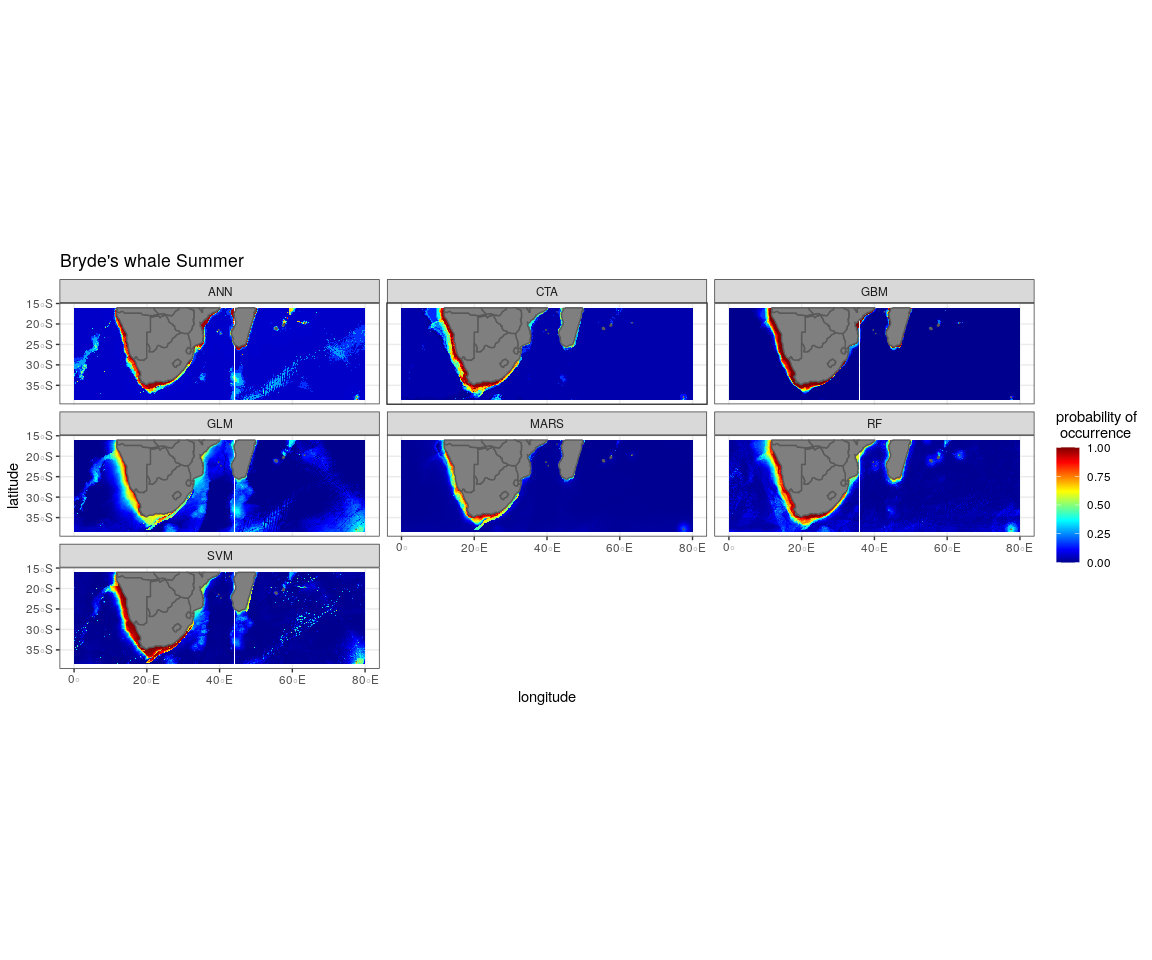

Supplement: Supplemental Information 17 [file peerj-08-9997-s017.png]

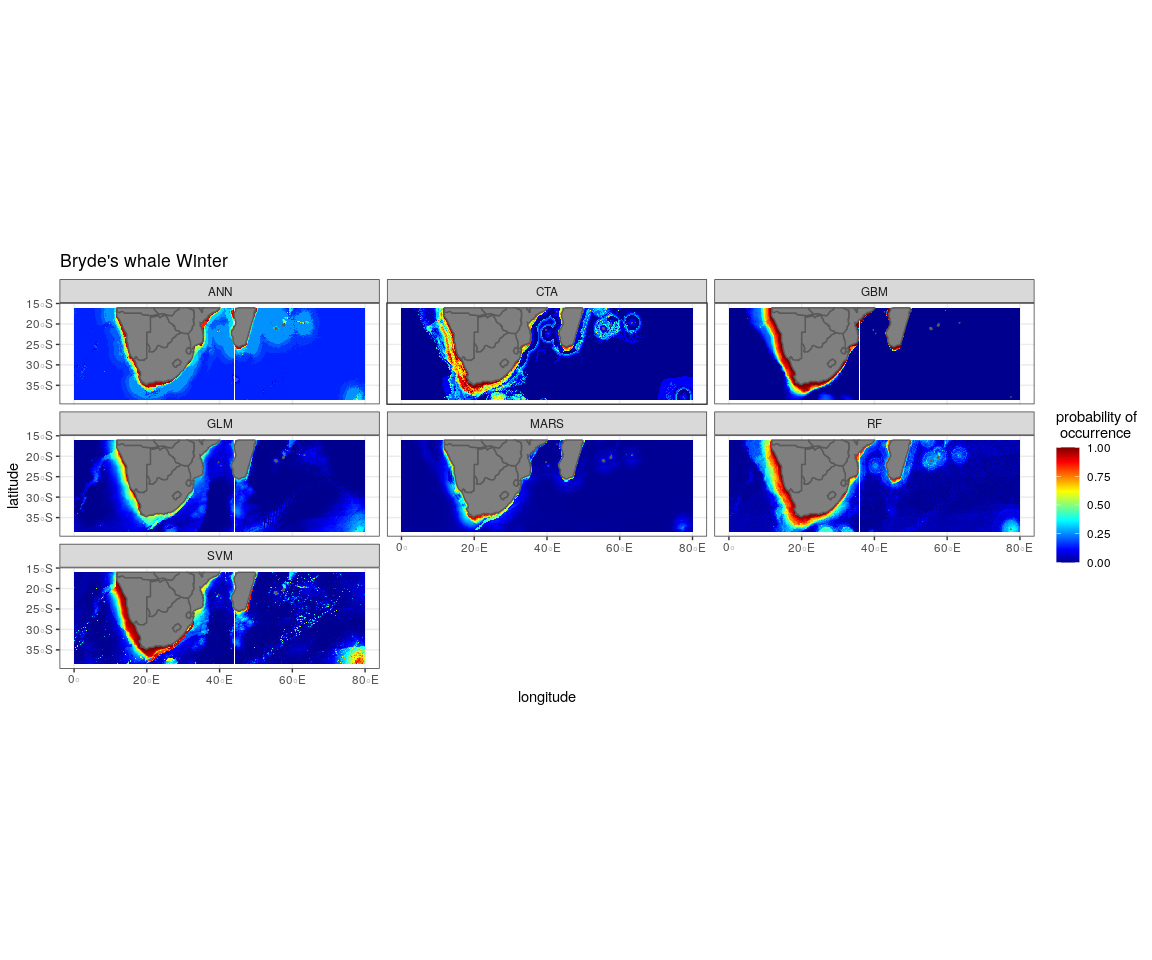

Supplement: Supplemental Information 18 [file peerj-08-9997-s018.png]

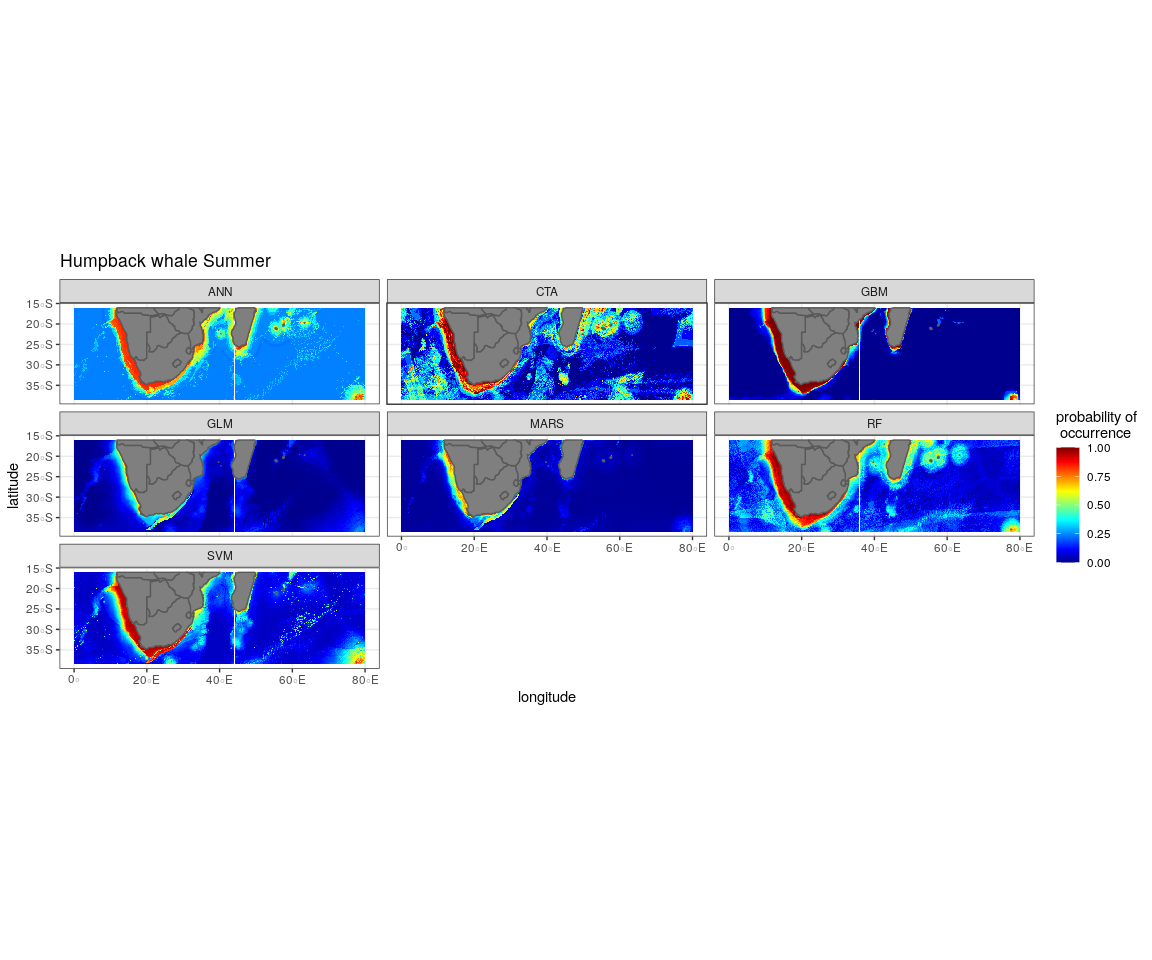

Supplement: Supplemental Information 19 [file peerj-08-9997-s019.png]

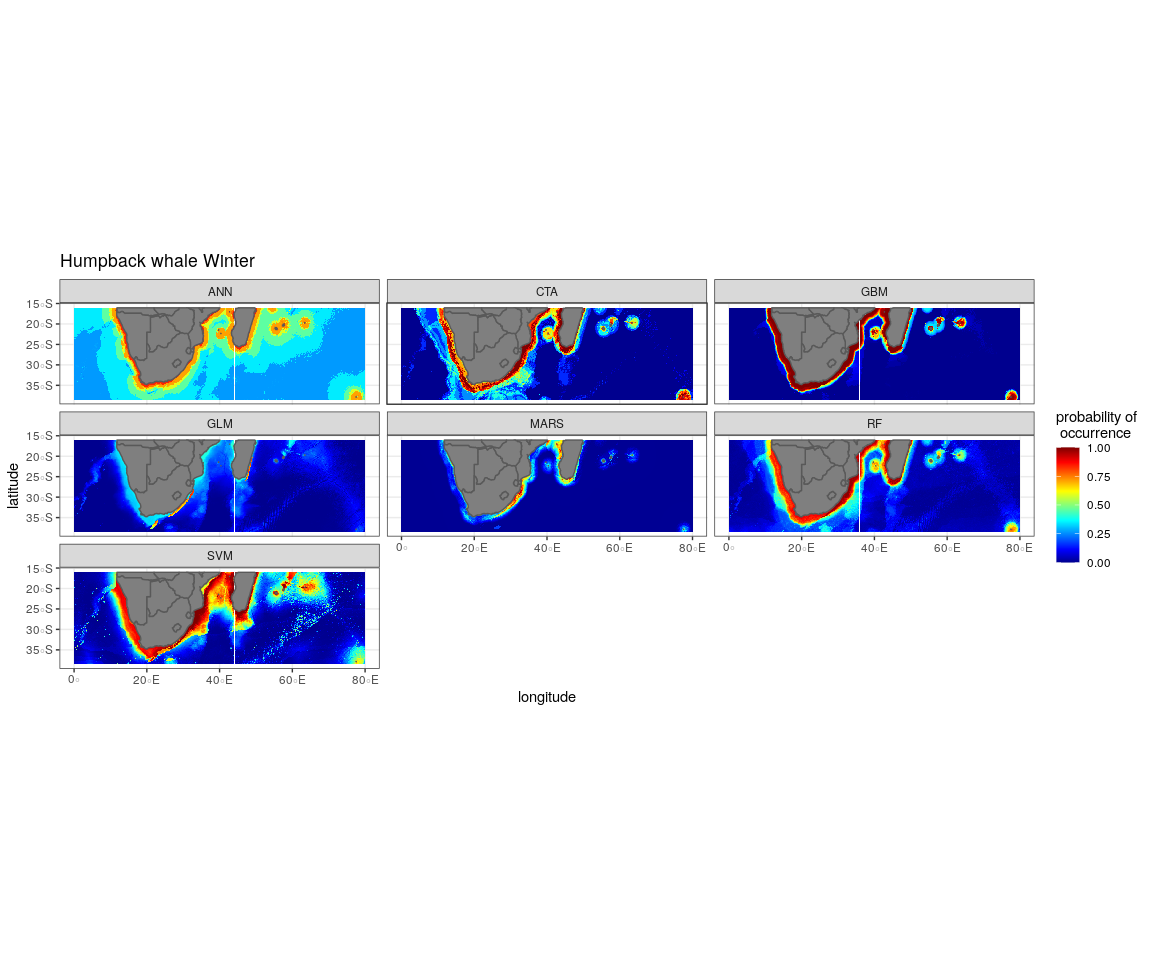

Supplement: Supplemental Information 20 [file peerj-08-9997-s020.png]

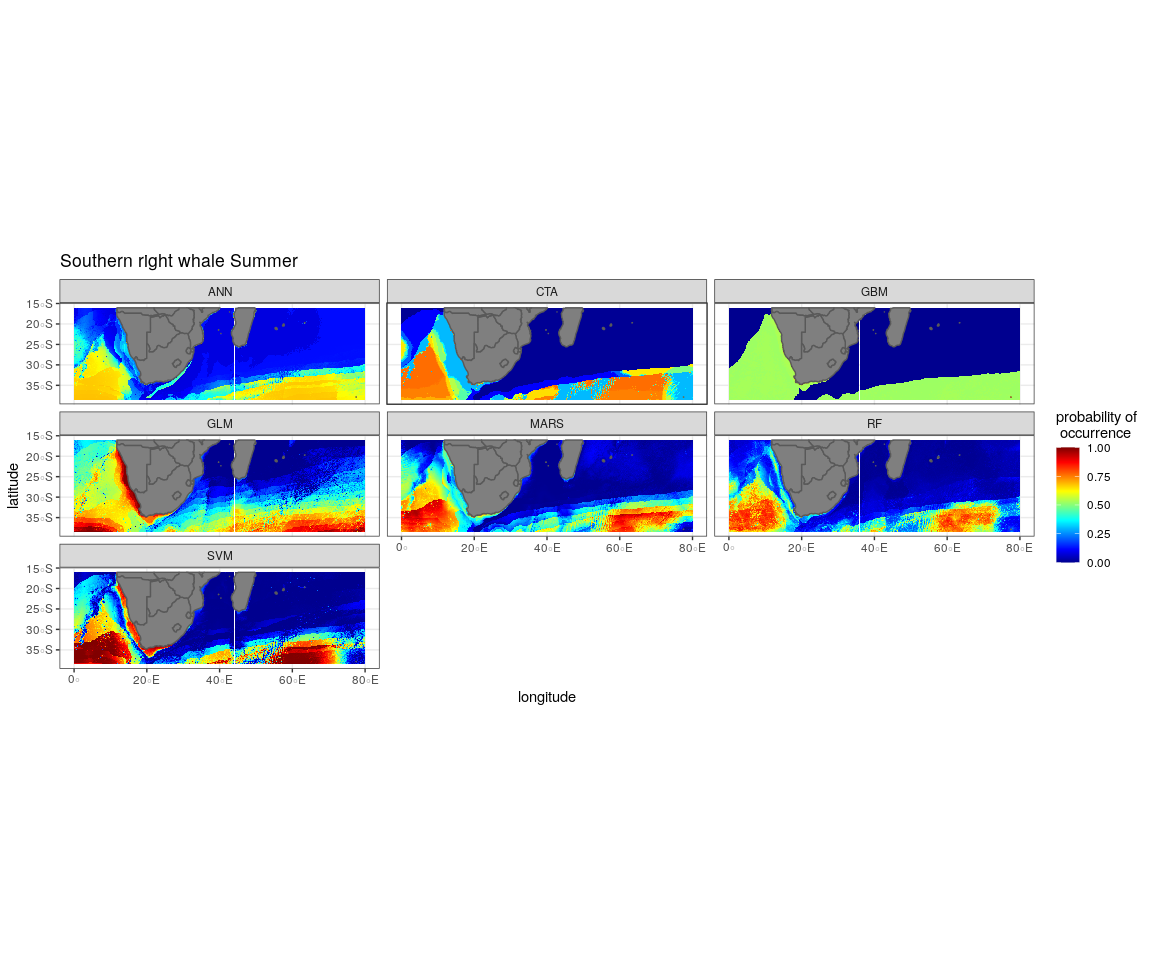

Supplement: Supplemental Information 21 [file peerj-08-9997-s021.png]

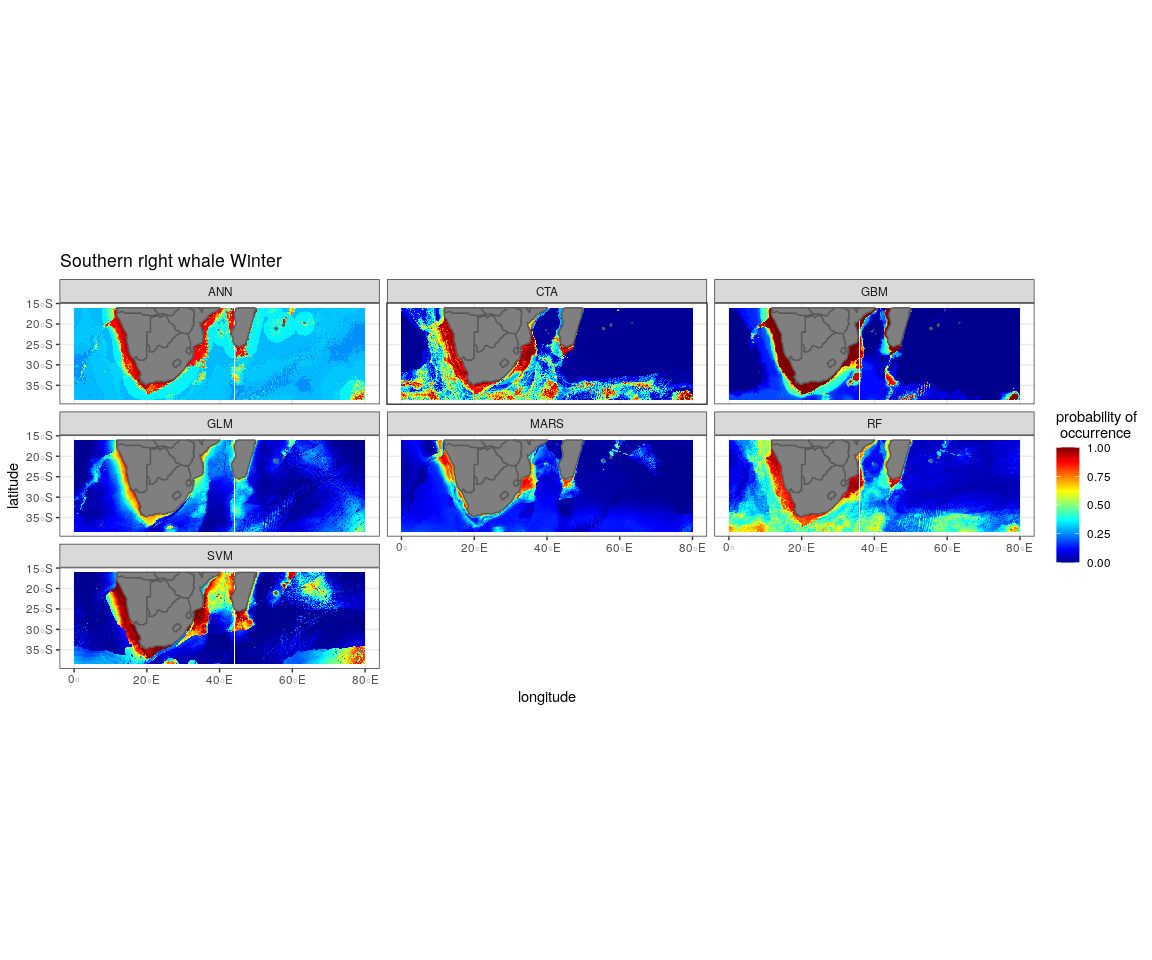

Supplement: Supplemental Information 22 [file peerj-08-9997-s022.png]

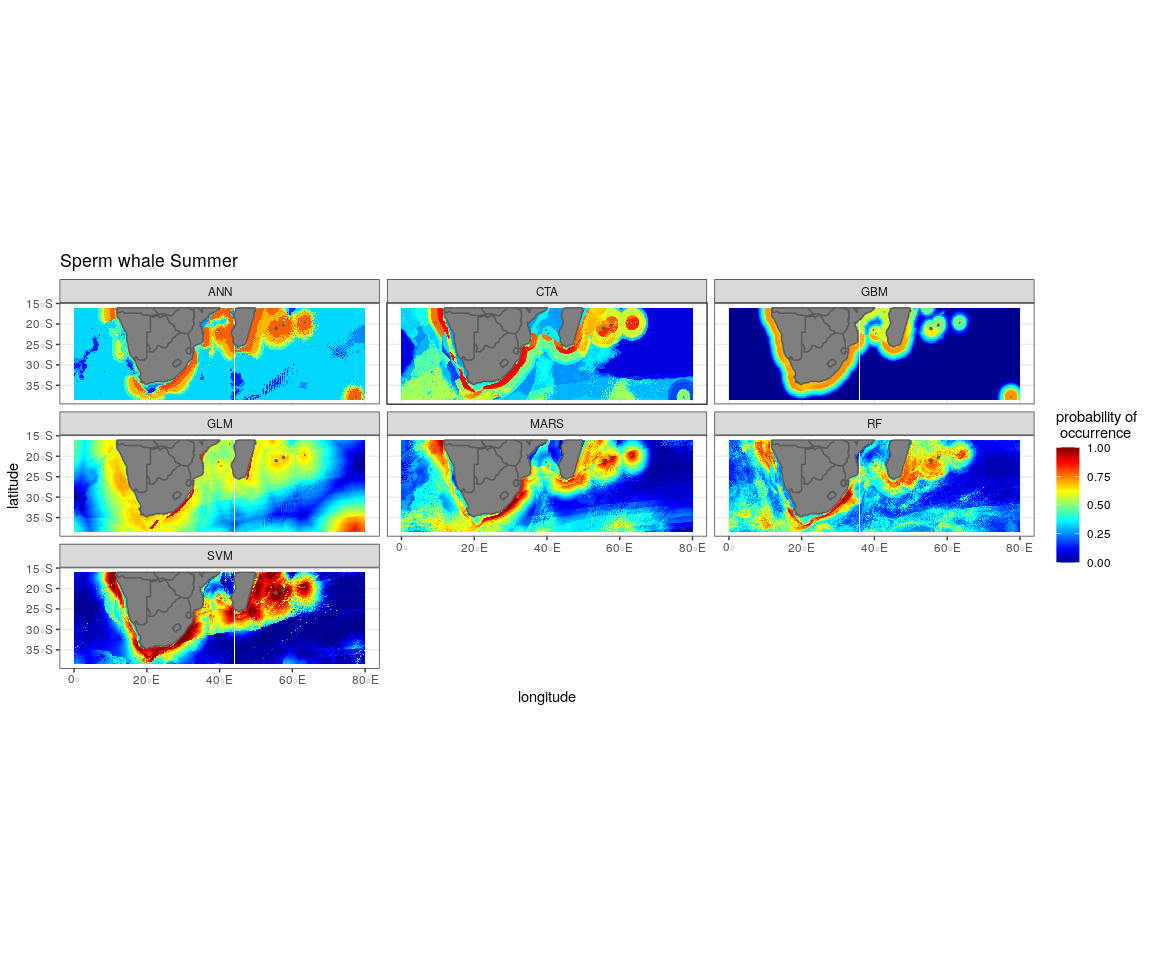

Supplement: Supplemental Information 23 [file peerj-08-9997-s023.png]

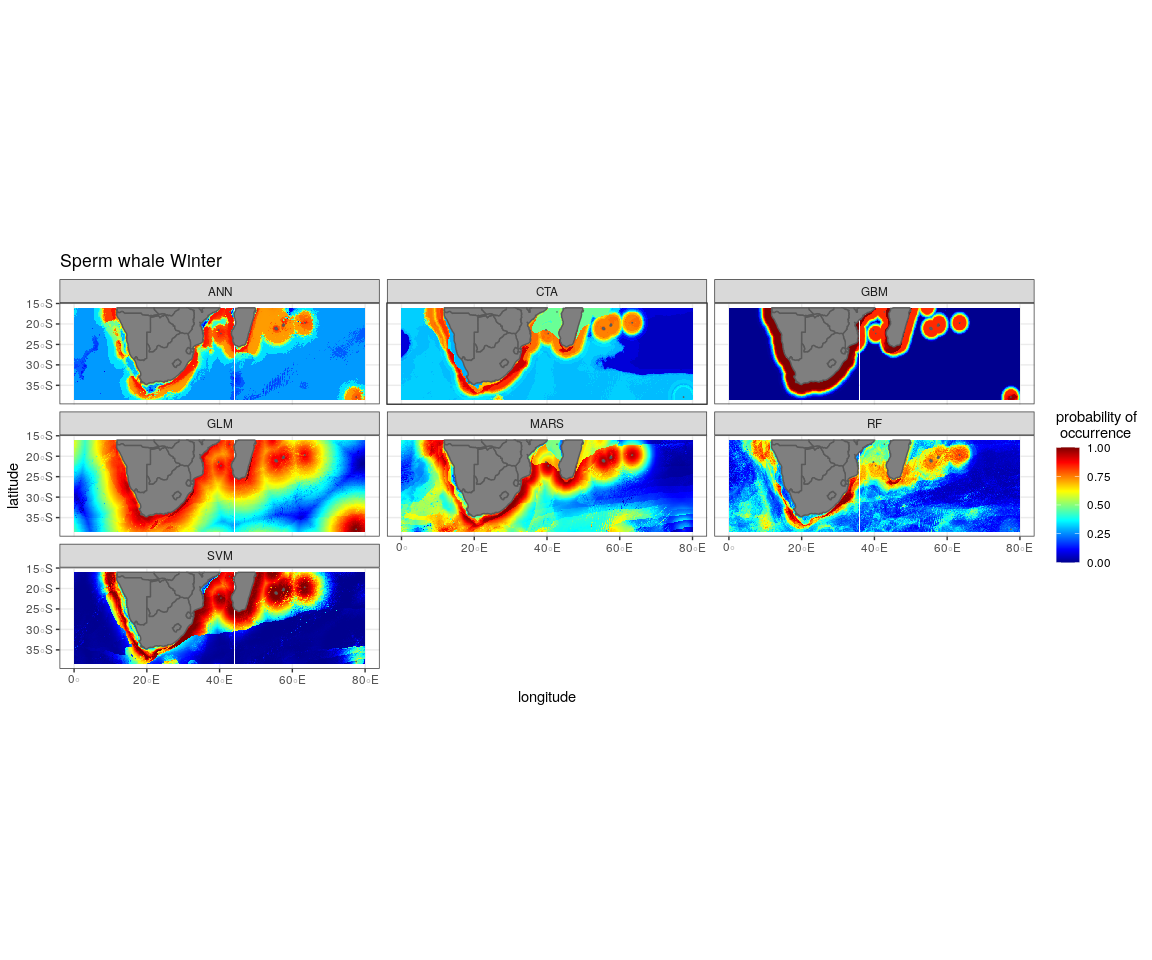

Supplement: Supplemental Information 24 [file peerj-08-9997-s024.png]
