# Supplementary material for: Species distribution modelling of Bryde’s whales, humpback whales, southern right whales, and sperm whales in the southern African region to inform their conservation in expanding economies
Source: PeerJ. 2020 Sep 22;8:e9997. doi: 10.7717/peerj.9997 (PMC7518163; doi:10.7717/peerj.9997)
Supplement: Table S1 [file peerj-08-9997-s025.pdf]

	Bryde's whale		Humpback whale		Southern right whale		Sperm whale		Grand Total
	**Summer**	**Winter**	**Summer**	**Winter**	**Summer**	**Winter**	**Summer**	**Winter**	
**Incidental sighting**	**154**	**235**	**7**	**7**	**2**	**4**	**-**	**-**	**409**
1965	-	-	-	1	-	-	-	-	**1**
1966	-	-	-	1	-	-	-	-	**1**
1971	-	-	-	-	1	-	-	-	**1**
1973	-	-	-	1	-	-	-	-	**1**
1975	5	-	-	-	-	-	-	-	**5**
1976	-	1	-	-	-	-	-	-	**1**
1977	1	2	1	-	1	-	-	-	**5**
1978	-	-	-	-	-	1	-	-	**1**
1980	-	-	-	-	-	2	-	-	**2**
1981	-	-	-	-	-	1	-	-	**1**
1982	-	7	-	-	-	-	-	-	**7**
1983	17	13	2	-	-	-	-	-	**32**
1984	6	6	2	1	-	-	-	-	**15**
1985	1	7	2	1	-	-	-	-	**11**
1986	7	3	-	-	-	-	-	-	**10**
1987	11	11	-	-	-	-	-	-	**22**
1988	10	16	-	-	-	-	-	-	**26**
1989	14	12	-	-	-	-	-	-	**26**
1990	8	12	-	-	-	-	-	-	**20**
1991	9	17	-	-	-	-	-	-	**26**
1992	2	8	-	-	-	-	-	-	**10**
1993	5	9	-	-	-	-	-	-	**14**
1994	9	5	-	1	-	-	-	-	**15**
1995	3	6	-	-	-	-	-	-	**9**
1996	3	3	-	-	-	-	-	-	**6**
1997	9	16	-	-	-	-	-	-	**25**
1998	6	19	-	1	-	-	-	-	**26**
1999	6	43	-	-	-	-	-	-	**49**
2000	-	2	-	-	-	-	-	-	**2**
2001	5	2	-	-	-	-	-	-	**7**

	Bryde's whale		Humpback whale		Southern right whale		Sperm whale		Grand Total
	**Summer**	**Winter**	**Summer**	**Winter**	**Summer**	**Winter**	**Summer**	**Winter**	
2002	6	10	-	-	-	-	-	-	**16**
2003	7	2	-	-	-	-	-	-	**9**
2004	4	3	-	-	-	-	-	-	**7**
**MMO**	**19**	**3**	**33**	**28**	**7**	**1**	**22**	**33**	**146**
2001	4	-	6	-	1	-	-	-	**11**
2004	-	-	2	-	-	-	-	-	**2**
2006	-	-	-	-	3	-	-	-	**3**
2007	7	-	10	-	-	-	-	-	**17**
2008	-	1	2	1	-	-	-	-	**4**
2009	-	-	4	-	-	-	-	-	**4**
2011	-	-	-	7	1	1	-	-	**9**
2012	4	2	2	-	-	-	-	-	**8**
2013	4	-	6	-	2	-	-	-	**12**
2014	-	-	1	-	-	-	-	-	**1**
2015	-	-	-	-	-	-	2	2	**4**
2016	-	-	-	20	-	-	20	31	**71**
**MRI**	**108**	**13**	**6**	**24**	**1143**	**41**	**297**	**1006**	**2638**
1963	10	13	-	3	2	-	40	142	**210**
1968	-	-	-	-	-	-	8	8	**16**
1969	-	-	-	-	-	-	-	8	**8**
1971	-	-	-	-	-	-	12	12	**24**
1972	-	-	-	5	-	-	42	215	**262**
1973	2	-	1	7	1	-	66	191	**268**
1974	-	-	-	4	-	1	45	205	**255**
1975	5	-	-	4	-	-	57	191	**257**
1977	-	-	-	-	1	-	-	-	**1**
1979	-	-	-	-	2	-	6	6	**14**
1981	-	-	-	-	-	-	-	1	**1**
1982	-	-	1	1	2	3	-	-	**7**
1983	84	-	3	-	1	-	-	-	**88**
1984	3	-	1	-	3	2	-	-	**9**

	Bryde's whale		Humpback whale		Southern right whale		Sperm whale		Grand Total
	Summer	Winter	Summer	Winter	Summer	Winter	Summer	Winter	
1985	-	-	-	-	2	-	1	1	**4**
1986	-	-	-	-	-	-	2	8	**10**
1987	-	-	-	-	-	-	10	10	**20**
1988	-	-	-	-	-	-	7	7	**14**
1992	1	-	-	-	-	-	1	1	**3**
1997	2	-	-	-	-	-	-	-	**2**
2013	-	-	-	-	416	-	-	-	**416**
2014	1	-	-	-	499	-	-	-	**500**
2015	-	-	-	-	214	35	-	-	**249**
**OBIS**	**3**	**3**	**39**	**23**	**891**	**195**	**654**	**1017**	**2825**
1913	-	-	36	19	889	186	631	985	**2746**
1966	-	-	-	-	-	-	-	1	**1**
1977	-	-	-	1	-	-	-	-	**1**
1978	-	-	-	-	-	1	-	-	**1**
1980	-	-	-	-	-	-	-	1	**1**
1981	-	-	-	-	-	-	-	1	**1**
1982	-	-	1	-	-	-	-	-	**1**
1983	1	-	-	-	-	-	-	-	**1**
1984	-	2	-	-	-	1	-	-	**3**
1985	-	-	-	-	-	-	1	1	**2**
1986	-	-	-	-	-	1	-	1	**2**
1987	-	-	-	-	-	2	-	-	**2**
1988	1	-	-	-	-	1	-	-	**2**
1989	1	-	-	-	-	-	-	-	**1**
1990	-	-	1	-	-	1	-	-	**2**
1991	-	-	-	-	-	-	-	1	**1**
1992	-	1	-	-	-	-	-	-	**1**
1994	-	-	-	-	2	2	-	-	**4**
1999	-	-	-	1	-	-	4	4	**9**
2003	-	-	-	1	-	-	2	2	**5**
2004	-	-	-	-	-	-	2	3	**5**

	Bryde's whale		Humpback whale		Southern right whale		Sperm whale		Grand Total
	Summer	Winter	Summer	Winter	Summer	Winter	Summer	Winter	
2006	-	-	1	1	-	-	-	-	**2**
2009	-	-	-	-	-	-	-	3	**3**
2010	-	-	-	-	-	-	14	14	**28**
**Grand Total**	**284**	**254**	**85**	**82**	**2043**	**241**	**973**	**2056**	**6018**

	Bryde's whale		Humpback whale		Southern right whale		Sperm whale		Grand Total
	Summer	Winter	Summer	Winter	Summer	Winter	Summer	Winter	
**Incidental sighting**	**154**	**235**	**7**	**7**	**2**	**4**			**409**
1965				1					**1**
1966				1					**1**
1971					1				**1**
1973				1					**1**
1975	5								**5**
1976		1							**1**
1977	1	2	1		1				**5**
1978						1			**1**
1980						2			**2**
1981						1			**1**
1982		7							**7**
1983	17	13	2						**32**
1984	6	6	2	1					**15**
1985	1	7	2	1					**11**
1986	7	3							**10**
1987	11	11							**22**
1988	10	16							**26**
1989	14	12							**26**
1990	8	12							**20**
1991	9	17							**26**
1992	2	8							**10**
1993	5	9							**14**
1994	9	5		1					**15**
1995	3	6							**9**

		Bryde's whale		Humpback whale		Southern right whale		Sperm whale		Grand Total
		Summer	Winter	Summer	Winter	Summer	Winter	Summer	Winter	
	1996	3	3							**6**
	1997	9	16							**25**
	1998	6	19		1					**26**
	1999	6	43							**49**
	2000		2							**2**
	2001	5	2							**7**
	2002	6	10							**16**
	2003	7	2							**9**
	2004	4	3							**7**
**MMO**		**19**	**3**	**33**	**28**	**7**	**1**	**22**	**33**	**146**
	2001	4		6		1				**11**
	2004			2						**2**
	2006					3				**3**
	2007	7		10						**17**
	2008		1	2	1					**4**
	2009			4						**4**
	2011				7	1	1			**9**
	2012	4	2	2						**8**
	2013	4		6		2				**12**
	2014			1						**1**
	2015							2	2	**4**
	2016				20			20	31	**71**
**MRI**		**108**	**13**	**6**	**24**	**1143**	**41**	**297**	**1006**	**2638**
	1963	10	13		3	2		40	142	**210**
	1968							8	8	**16**
	1969								8	**8**
	1971							12	12	**24**
	1972				5			42	215	**262**
	1973	2		1	7	1		66	191	**268**
	1974				4		1	45	205	**255**
	1975	5			4			57	191	**257**

		Bryde's whale		Humpback whale		Southern right whale		Sperm whale		Grand Total
		Summer	Winter	Summer	Winter	Summer	Winter	Summer	Winter	
	1977					1				**1**
	1979					2		6	6	**14**
	1981								1	**1**
	1982			1	1	2	3			**7**
	1983	84		3		1				**88**
	1984	3		1		3	2			**9**
	1985					2		1	1	**4**
	1986							2	8	**10**
	1987							10	10	**20**
	1988							7	7	**14**
	1992	1						1	1	**3**
	1997	2								**2**
	2013					416				**416**
	2014	1				499				**500**
	2015					214	35			**249**
**OBIS**		**3**	**3**	**39**	**23**	**891**	**195**	**654**	**1017**	**2825**
	1913			36	19	889	186	631	985	**2746**
	1966								1	**1**
	1977				1					**1**
	1978						1			**1**
	1980								1	**1**
	1981								1	**1**
	1982			1						**1**
	1983	1								**1**
	1984		2				1			**3**
	1985							1	1	**2**
	1986						1		1	**2**
	1987						2			**2**
	1988	1					1			**2**
	1989	1								**1**
	1990			1			1			**2**

	Bryde's whale		Humpback whale		Southern right whale		Sperm whale		Grand Total
	Summer	Winter	Summer	Winter	Summer	Winter	Summer	Winter	
1991								1	**1**
1992		1							**1**
1994					2	2			**4**
1999				1			4	4	**9**
2003				1			2	2	**5**
2004							2	3	**5**
2006			1	1					**2**
2009								3	**3**
2010							14	14	**28**
**Grand Total**	**284**	**254**	**85**	**82**	**2043**	**241**	**973**	**2056**	**6018**
