# Supplementary material for: Species distribution modelling of Bryde’s whales, humpback whales, southern right whales, and sperm whales in the southern African region to inform their conservation in expanding economies
Source: PeerJ. 2020 Sep 22;8:e9997. doi: 10.7717/peerj.9997 (PMC7518163; doi:10.7717/peerj.9997)
Supplement: Table S2 [file peerj-08-9997-s026.docx]

|  | **Bryde's whale** | | **Humpback whale** | | **Southern right whale** | | **Sperm whale** | |
| --- | --- | --- | --- | --- | --- | --- | --- | --- |
|  | **Summer** | **Winter** | **Summer** | **Winter** | **Summer** | **Winter** | **Summer** | **Winter** |
| **EM** | 0.96 | 0.95 | 0.87 | 0.92 | 0.88 | 0.85 | 0.77 | 0.82 |
| **ANN** | 0.93 | 0.9 | 0.78 | 0.85 | 0.79 | 0.77 | 0.72 | 0.76 |
| **CTA** | 0.94 | 0.92 | 0.81 | 0.88 | 0.9 | 0.81 | 0.78 | 0.82 |
| **GBM** | 0.97 | 0.98 | 0.91 | 0.95 | 0.87 | 0.87 | 0.75 | 0.82 |
| **GLM** | 0.98 | 0.96 | 0.9 | 0.94 | 0.85 | 0.84 | 0.72 | 0.78 |
| **MARS** | 0.97 | 0.97 | 0.89 | 0.92 | 0.92 | 0.91 | 0.81 | 0.84 |
| **RF** | 0.98 | 0.97 | 0.92 | 0.97 | 0.92 | 0.92 | 0.84 | 0.87 |
| **SVM** | 0.98 | 0.95 | 0.89 | 0.93 | 0.89 | 0.81 | 0.77 | 0.82 |
